# Hypersensitivity reactions to biologics (part I): allergy as an important differential diagnosis in complex immune-derived adverse events

**DOI:** 10.1007/s40629-020-00126-6

**Published:** 2020-05-12

**Authors:** Askin Gülsen, Bettina Wedi, Uta Jappe

**Affiliations:** 1 Division of Clinical and Molecular Allergology, Airway Research Center North (ARCN), Member of the German Center for Lung Research, Research Center Borstel, Parkallee 35, 23845 Borstel, Germany; 2 Interdisciplinary Allergy Outpatient Clinic, Department of Pneumology, University of Luebeck, Luebeck, Germany; 3 Department of Dermatology and Allergy, Comprehensive Allergy Centre, Hannover Medical School, 30625 Hannover, Germany

**Keywords:** Adverse drug reaction, Anaphylaxis, Anti-drug antibodies, Biologicals, Infusion reactions

## Abstract

**Purpose:**

Biotechnological substances (BSs) are strongly relied upon to prevent rejection of transplanted organs, and to treat oncological, allergological, and other inflammatory diseases. Allergic reactions to partly foreign biologics can occur due to their potential immunogenicity. The severity of an immune response to a biological drug may range from no clinical significance to a severe, life-threatening anaphylactic reaction.

**Methods:**

Detailed searches were performed on Pubmed, Web of Science, and Google Scholar to include all available publications. In addition, the Food and Drug Administration, the European Medicines Agency, and British Columbia Cancer Agency Drug Manual databases were screened for hypersensitivity reaction (HSR), infusion reaction, injection site reaction, urticaria, and anaphylaxis for individual BSs.

**Results:**

Treatment with BSs can cause various types of HSR. These are mentioned in the literature with definitions such as allergic reactions, anaphylactoid reactions, anaphylaxis, HSR, infusion reactions, injection site reactions, cytokine release syndrome, and urticaria. Due to the overlap in signs and symptoms in the reported descriptions, it is not always possible to differentiate these reactions properly according to their pathomechanism. Similarly, many data reported as anaphylaxis actually describe severe anaphylactic reactions (grades III or IV).

**Conclusion:**

There is an urgent need for a simpler symptom- or system-based classification and scoring system to create an awareness for HSRs to BSs. A better understanding of the pathophysiology of HSRs and increased clinical experience in the treatment of side effects will provide timely control of unexpected reactions. As a result, immunotherapy with BSs will become safer in the future.

## Introduction

Biotechnological substances (BSs), also known as biological agents, biologicals or biologics, are produced by living organisms or are synthesized from a product made by a living organism. Some BSs are directed against specific cytokines or cell surface signal receptors. In recent years, the BS industry has experienced rapid growth and yielded products for treatment of a broad spectrum of diseases and disorders. Biologics are strongly relied upon to prevent rejection after organ transplantation and to treat diseases related to allergology, pneumology, rheumatology, oncology, and dermatology. BSs have revolutionized the treatment of diseases such as rheumatoid arthritis, psoriatic arthritis, inflammatory bowel disease, and organ-specific cancers (lung, stomach, colon, cervix, breast, etc.). Other conditions commonly treated or managed with biologics include several types of asthma, plaque psoriasis, chronic urticaria, and vasculitis. Some new indications and less studied applications include interstitial lung disease, unstable angina pectoris during cardiac catheterization, paroxysmal nocturnal hemoglobinuria, neonatal bronchopulmonary dysplasia, and multiple sclerosis (Table 1).

**Table 1:** Overview of some biotechnological substances to date (February 2020)

Department	Generic	Brands®	Target	Primary indications
Pneumology	Benralizumab	Fasenra®	IL‑5 receptor (CD125)	Asthma
	Lebrikizumab	–	IL-13	Asthma, atopic dermatitis
	Mepolizumab	Nucala®	IL‑5	Asthma
	Reslizumab	Cinqaero, Cinqair®	IL‑5	Asthma
	Omalizumab	Xolair®	IgE	Asthma, urticaria
	Nintedanib	OFEV® Vargatef®	Tyrosine kinase inhibitor VEGFR, FGFR, PDGFR	Idiopathic pulmonary fibrosis, non-small cell lung carcinoma
	Pirfenidon	Esbriet® Pirespa®	Transforming growth factor beta (TGF-β)	Idiopathic pulmonary fibrosis
Dermatology	Alefacept	Amevive®	CD2	Psoriasis
	Efalizumab	Raptiva®	CD11a	Psoriasis
	Ixekizumab	Taltz®	IL-17a	Psoriasis
	Secukinumab	Cosentyx®	IL-17a	Psoriasis, psoriatic arthritis, ankylosing spondylitis
	Ustekinumab	Stelara®	IL-12/IL-23	Psoriasis
	Dupilumab	Dupixent®	IL-4/IL-13	Atopic dermatitis, asthma
	Ligelizumab	–	IgE	Chronic urticaria
	Quilizumab	–	Membrane-expressed IgE	Chronic urticaria, asthma
Oncology	Aflibercept	Zaltrap®, Eylea®	VEGF‑1	Colorectal carcinom, macula degeneration
	Alemtuzumab	Campath® Lemtrada®	CD52	Leukemia, multiple sclerosis
	Atezolizumab	Tecentriq®	PD-L1	SCLC, NSCLC, breast cancers, prostate cancer
	Bevacizumab	Avastin® Mvasi® Zirabev®	CD11a	Bowel, lung, kidney, cervix, breast cancers
	Bilinatumomab	Blincyto®	CD19 (B-cells), CD3 (T-cells)	ALL
	Cetuximab	Erbitux®	EGFR (HER1/neu, ErbB1)	Colorectal carcinoma, Squamous cell carcinoma of the head and neck area
	Durvalumab	Imfinzi®	PD-L1	NSCLC, urothelial carcinoma
	Gemtuzumab ozogamicin	Mylotarg®	CD33	AML
	Ibritumomab tiuxetan	Zevalin®	CD20	Lymphoma
	Imatinib	Gleevec® Glivec®	Tyrosinkinase inhibitor	Leukemia, GI tumors Mastocytosis
	Ipilimumab	Yervoy®	CTLA‑4	Melanoma, renal cell carcinoma Colorectal carcinoma
	Necitumumab	Portrazza®	EGFR	NSCLC
	Nivolumab	Opdivo®	PD‑1 receptor	Melanoma, HCC, NSCLC, Hodgkin’s lymphoma, renal cell carcinoma, urothelial cancer
	Panitumumab	Vectibix®	EGFR	Colorectal carcinoma
	Pembrolizumab	Keytruda®	PD‑1 receptor	NSCLC, Melanoma, Lymphoma
	Pertuzumab	Perjeta®	EGFR (HER2)	Breast carcinoma
	Ramucirumab	Cyramza®	VEGF‑2	NSCLC, HCC, colorectal and gastric carcinoma
	Trastuzumab	Herceptin, Kadycla®	HER2/neu, ErbB2 (EGFR)	Breast, bowel, gastric carcinoma
Rheumatology	Adalimumab	Humira®	TNF‑α	RA, psoriatic arthritis, Crohn’s disease, colitis ulcerosa
	Anakinra	Kineret®	IL‑1 receptor	RA, CAPS, Still’s disease
	Belimumab	Benlysta®	BLyS	SLE
	Canakinumab	Ilaris®	IL‑1 β	CAPS, Still’s disease, autoinflammatory recurrent fever syndromes, TRAPS
	Etanercept	Enbrel®, Erelzi®	TNF‑α-RII	RA, psoriatic arthritis
	Golimumab	Simponi®	TNF‑α	RA
	Infliximab	Remicade®	TNF‑α	RA, colitis ulcerosa, psoriatic arthritis, ankylosing spondylitis
	Certolizumab	Cimzia®	TNF‑α	Crohn’s disease, RA, psoriatic arthritis
	Rituximab	Rituxan® MabThera®	CD20	RA, MPA, lymphoma, leukemia
Tranplantation	Basiliximab	Simulect®	IL-2R	Kidney transplantation
	Belatacept	Nulojix®	CD80/CD86	Kidney transplantation
	Daclizumab	Zenapax® Zinbryta®	IL-2R α	Kidney transplantation, multiple sclerosis
	Muromonab	Orthoclone OKT3®	CD3	Kidney, liver, heart transplantation
Various	Abciximab	ReoPro®	Platelet GP IIb/IIIa	Unstable angina while engaging cardiac catheter
	Eculizumab	Soliris ®	Complement C5	PNH, atypical haemolytic uremic syndrome
	Lanadelumab	Takhzyro®	Plasma kallikrein	Hereditary angioedema
	Natalizumab	Tysabri®	Integrin a4	Multiple sclerosis
	Palivizumab	Synagis®	F‑protein of RSV	Bronchopulmonary dysplasia, congenital heart disease

*ALL* acute lymphoblastic leukemia, *AML* acute myeloid leukomia, *BlyS* B-lymphocyte stimulator, *CAPS* cryopyrine associated periodic syndrome, *CD* cluster of differentiation, *CTLA‑4* cytotoxic T‑lymphocyte antigen‑4, *EGFR* epidermal growth factor receptor, *FGFR* fibroblast growth factor receptor, *GI* gastrointestinal, *HCC* hepatocellular carcinoma, *IL* interleukin, *MPA* microscopic polyangiitis, *NSCLC* non-small cell lung cancer, *PD-L1* programmed cell death ligand 1, *PDGFR* platelet derived growth factor receptor, *PNH* paroxysmal nocturnal hemoglobinuria, *RA* rheumatoid arthritis, *RSV* respiratory syncytial virus, *SCLC* small cell lung cancer, *SLE* systemic lupus erythematosus, *TNF* tumor necrosis factor, *TRAPS* TNFR1-associated periodic fever syndrome, *VEGF* vascular endothelial growth factor, *VEGFR* vascular endothelial growth factor receptor

Allergic reactions to partly foreign biologics can occur due to their potential immunogenicity. Adverse drug reactions (ADRs), however, can also result from an agent’s direct biological function. For instance, neutralization of an off-target cytokine’s activity can cause ADRs [1]. The severity of an immune response to a biological drug may range from no clinical significance to a severe, life-threatening anaphylactic reaction [2]. Thus it became evident that the nomenclature used to describe and document immunological adverse events was not always precise enough, or the cases had not been investigated in enough detail to clearly differentiate between truly allergic and other reactions. This review will evaluate reports of allergic and substance-specific infusion reactions (IR), injection-site reactions (ISR), hypersensitivity reactions (HSR), urticaria, and anaphylaxis caused by BSs.

### Material and methods

BSs were investigated individually for HSR, IR, ISRs, and anaphylaxis from The Food and Drug Administration (FDA), European Medicines Agency (EMA), and British Columbia Cancer Agency (BCCA) Drug Manual database and created the main information pool in terms of these side effects. In addition, case reports, articles, and reviews on this subject were retrieved by searching three international databases (Pubmed, Web of Science, and Google Scholar). The search terms, such as specific BS, allergy, anaphylaxis, hypersensitivity, IR, ISR, and urticaria were determined for the search.

### Overview of reported allergic reactions to biotechnological substances

BSs are engineered macromolecules that are quite similar to autologous proteins. Therefore, they vary, as do small-molecule pharmaceuticals, in the reactions they produce. In a comprehensive review of the Spanish Society of Rheumatology’s Biobadaser Database in 2018, 8253 side effects were reported in 4454 patients treated with BSs (mostly tumor necrosis factor [TNF]-α-blockers) [3]. Of these, 7133 (86.4%) were classified as non-serious, 1089 (13.2%) as serious, and 31 (0.38%) as fatal.

The symptoms mentioned in the various classifications of HSRs and anaphylaxis have many common features. Due to the overlap in these common signs and symptoms, it is not possible to differentiate between IRs, anaphylaxis, or other HSRs in the reported descriptions and publications. In this review, we will elaborate IR, ISR, HSR, urticaria, as well as anaphylaxis. Drugs are described individually under subheadings naming the various medical disciplines in which they are used.

## Pulmonology

The most common indications for the use of biologics in lung diseases are allergic and severe uncontrolled asthma. Biologics are used more rarely in interstitial lung diseases (Table 2). Their use in lung cancer will be mentioned in the section *Oncology*. Benralizumab, lebrikizumab, mepolizumab, reslizumab, and omalizumab are used in asthmatic patients. The primary indication for nintedanib and pirfenidone is idiopathic pulmonary fibrosis.

**Table 2:** Reported allergic reactions to biotechnological substances (Pulmonology)

Biologics	ROA	Feature	Authors	Year	HSR %	IR %	ISR %	Urticaria %	Anaphylaxis %
Benralizumab	s.c.	Humanized	Castro et al. [4]	2014	–	–	16.0	–	0
			FDA [5]	2017	3.0		2.2	–	–
			Park et al. [6]	2019	–		0	0–2.0	–
			Pelaia et al. [7]	2019	0		–	–	0
			Liu et al. [8]	2019	–		2.6	–	–
Lebrikizumab	s.c.	Humanized	Hanania et al. [9]	2015	0–0.9	–	11.1–20.5	–	–
			Hanania et al. [10]	2016	–		6.0–10.0	–	<1.0
			Korenblat et al. [11]	2018	–		2.9	–	1.0
			Simpson et al. [12]	2018	–		1.3	–	0
Mepolizumab	i.v. s.c.	Humanized	Pavord et al. [13]	2012	0–1.0	5–12	–	–	0
			FDA [14]	2015	–	–	3.0–8.0	–	0
			Lugogo et al. [15]	2016	<1	<1.0	3.0	–	0
			Khatri et al. [16]	2019	2.0	–	12.0	–	0
Reslizumab	i.v.	Humanized	Castro et al. [17]	2015	–	–	2.0	–	<1
			FDA [18]	2016	–	–		–	<1
			Murphy et al. [19]	2017	<1	<1		<1	0
Omalizumab	s.c.	Humanized	Cox et al. [21]	2007	<0.2	–	–	–	0.09
			Di Bona et al. [22]	2017	–		3.4	1.0	0
			FDA [24]	2019	–		12.0–45.0	0.2	0.1

*ROA* route of administration, *HSR* hypersensitivity reaction, *IR* infusion reaction, *ISR* Injection-site reaction

### Benralizumab

Benralizumab is the newest anti-asthmatic and anti-inflammatory drug in the monoclonal antibody (mAb) family developed for the treatment of severe eosinophilic and allergic asthma. It exerts its effects by binding to the α‑subunit of the interleukin (IL)-5 receptor on eosinophils and basophils.

In a phase IIb study conducted between 2011 and 2012, the ISR rate was 16% and anaphylaxis was 0% [4]. FDA labels show a 3% incidence of HSRs (anaphylaxis, urticaria, rash, and angioedema) in the treatment group receiving benralizumab and placebo [5]. The frequency of ISR (erythema, local pain, papule, or pruritus) was 2.2% in patients treated with benralizumab [5]. These reactions usually occurred within the first hours after s.c. administration of the drug. The most reported ADR was upper respiratory tract infection and mild to moderate nasopharyngitis [6]. In a recent study in which 13 patients were treated, HSR and anaphylaxis were not reported; only slight fever with chills was observed in two patients [7].

In the meta-analysis of eight randomized controlled trials, the overall risk of ADRs and severe side effects was lower and the risk of headache and pyrexia higher in patients treated with benralizumab compared to placebo [8]. In addition, an increased incidence of ISRs, hypersensitivity, and death was not observed compared to placebo. The ISRs were reported in 2.6% of patients treated with 30 mg benralizumab. Post-marketing side effect recording is ongoing.

### Lebrikizumab

Lebrikizumab is a novel humanized mAb that specifically inhibits the activity of IL-13. Clinical studies for the treatment of asthma that is uncontrolled by inhaled corticosteroids, chronic obstructive pulmonary disease (COPD), atopic dermatitis, and idiopathic pulmonary fibrosis (IPF) are ongoing.

In one placebo-controlled study using higher doses, ISRs (pain, erythema) were found to be more prevalent, 20.5% and 20.3% in groups receiving 125 and 250 mg doses of lebrikizumab, 11.1% in a group receiving 37.5 mg, and 6% in the group given placebo [9]. In the phase III study (LAVOLTA) of lebrikizumab for asthma, ISRs occurred in 6% of participants receiving 37.5 mg s.c. injections, 10% of participants receiving 125 mg s.c. injections, and 8% of participants receiving a placebo once every 4 weeks [10]. In the same study, anaphylaxis was reported to be below 1%.

Korenblat et al. reported anaphylaxis in one patient (1%) treated with lebrikizumab, and this reaction was attributed to a known peanut allergy [11]. In another study, lebrikizumab was well tolerated, and death and anaphylactic reactions were not reported [12]. ISRs rarely occurred (1.3% in the lebrikizumab and 1.9% in the placebo group).

### Mepolizumab

Mepolizumab is a comparatively new anti-inflammatory, anti-asthmatic BS consisting of a mAb that binds to IL‑5. Therefore, it was also approved by the FDA in December 2017 for use in the treatment of Churg-Strauss syndrome (eosinophilic granulomatosis with polyangiitis).

In a multicenter study covering the years 2009–2011 (DREAM study), the most commonly reported drug-related adverse events were non-allergic, infusion-related reactions [13]. Infusion-related reactions occurred in 5% of participants receiving 75 mg of mepolizumab, 8% of participants receiving 250 mg, 12% of participants receiving 750 mg, and 6% of participants receiving placebo. HSRs thought to be associated with the study drug occurred in 0% of participants receiving 75 mg mepolizumab, <1% of participants receiving 250 mg, 1% of participants receiving 750 mg, and 2% of participants receiving placebo. No anaphylactic reaction was reported.

Anaphylaxis has not been reported on FDA labels [14]. However, in one case, type-IV delayed HSR was observed in the 9th month of treatment, 3 days after subcutaneous (SC) administration of 100 mg mepolizumab. Local ISRs (8% after 100 mg application, 3% after 75 mg application, 3% after placebo), and only few allergic reactions (≤2%) have been reported.

Lugogo et al. [15] conducted a study on SC administration of mepolizumab in severe eosinophilic asthma patients. In this study, HSRs (type IV) occurred in ≤1% of participants, local ISRs occurred in 3%, and <1% of participants experienced injection-related reactions (non-allergic). There was no report of mepolizumab-related anaphylaxis.

Finally, in a recent study of 347 severe eosinophilic asthma patients, local ISRs (12%), allergic/HSRs (2%), and non-allergic systemic reactions (<1%) were reported [16]. Anaphylaxis was not observed.

### Reslizumab

Reslizumab is a biologically active humanized mAb used for adjunctive therapy of severe asthma with an eosinophilic phenotype. This drug binds and inhibits the cytokine IL‑5, which is responsible for growth, differentiation, recruitment, activation, and survival of eosinophils.

In a study conducted by Castro et al. [17] in 2015, ISRs (hematoma, rash, and local pain) were reported to be less than 2%, and an anaphylactic reaction was reported in two patients. Anti-drug antibodies (ADA) for reslizumab were found to be negative in these patients, and therefore no further information could be obtained.

In FDA labels, treatment-related anaphylaxis was reported in three patients in the reslizumab 3 mg/kg treatment group [18]. The same label states that this drug is produced in the murine cell line, which introduces the galactose-α‑1,3‑galactose (α-gal) carbohydrate sequence as a post-translational modification into the carbohydrate side chains of the mAb. This explains why this drug is immunogenic for humans, but it is unclear how α‑gal acts and which role it plays in these reactions. ADA were positive in approximately 6% of patients treated with reslizumab, but there was no correlation with HSR and allergic reactions.

Murphy and colleagues [19] investigated the long-term effects of reslizumab and reported two HSRs (<1%), two drug eruptions (<1%), and very rare local infusion-related AEs (e.g., pain at injection site) (<1%). They did not report anaphylaxis during the follow-up period.

In a recent study, one case of toxicoderma (the 4th dose of the drug 12 h after administration) was reported [20]. The patient developed a symmetrical rash on the trunk and proximal limbs, and the symptoms improved after administration of systemic corticosteroids and antihistamines.

### Omalizumab

Omalizumab is a humanized mAb commonly used as an adjunctive and second-line treatment for severe allergic asthma and chronic spontaneous urticaria. The effect is due to selective binding to immunoglobulin E (IgE).

The task force report published by the American Allergy Academy in 2007 states that the rate of anaphylaxis associated with omalizumab treatment is 0.09% [21]. Of the reactions observed to date, 61% occurred within 2 h after one of the first three doses, and 14% occurred within 30 min after the fourth or a later dose. Omalizumab is produced in the ovarian cells of a Chinese hamster and does not express the α‑1,3‑galactosyl transferase, and therefore does not contain α‑gal. As a consequence, pre-existing IgE-antibodies as have been described in patients with anaphylaxis to cetuximab were not detectable in humans [21].

In a large-scale real life study conducted between 2007 and 2016, ISRs, immediate local reactions, urticaria, and anaphylaxis were reported to be 3.4%, 1.1%, 1.0%, and 0%, respectively [22].

In 2011, a sequel of the task force report of the American Allergy Academy written in 2007 was published on the basis of post-marketing safety data provided by Genentech/Novartis [23]. In this additional report, a total of 77 of the 127 patients (60.6%) that developed anaphylaxis against omalizumab reported that these reactions occurred within the recommended hospital waiting period (especially within the first 2 h after the first three injections).

In FDA labels, anaphylaxis with symptoms such as angioedema, bronchospasm, urticaria, hypotension, and syncope has been reported in 0.1% of patients [24]. This can be seen after the first dose, as well as 1 year later. ISRs of any severity (pain, burning, induration, warmth, redness, bruising, itching, granuloma formation, etc.) were observed in 45% of patients compared with placebo (43%). More severe reactions have been reported in 12% of patients receiving omalizumab and 9% in placebo. Most of these reactions occur 1 h after injection and last for less than 8 days.

### Nintedanib

Nintedanib is an antiproliferative and antitumoral oral BS of the multikinase receptor inhibitor group usually used in the treatment of idiopathic pulmonary fibrosis and non-small cell lung cancer.

The Australian Public Assessment report and the FDA label did not observe an increase in the incidence of severe immunological and anaphylactic reactions related to the use of nintedanib [25, 26]. Both reports documented hepatotoxicity, bleeding and thromboembolic events, gastrointestinal toxicity (diarrhea, nausea, and vomiting) and perforation, myocardial infarction, and hypertension as side effects. In post-marketing studies, it is reported that pruritus and rash may be seen, but the rate is not clear (referenced in [26]). Nintedanib capsules contain the following excipients; *(i) capsule content:* triglycerides, hard fat, lecithin (soya); *(ii) capsule shell:* gelatin, glycerol, titanium dioxide, iron oxide red and yellow; *(iii) printing ink:* shellac glaze, iron oxide black, and propylene glycol [26]. In addition, the summary of product characterics indicate that those patients with soy and peanut allergy should be treated with caution, but more detailed information as to the reason for legume allergy to be considered as a risk is lacking.

Insufficient data were found in our literature review to assess the prevalence of allergic reactions, HSR, anaphylaxis, and urticaria due to the use of this BS. This is probably due to the fact that other side effects were considered as having higher priority.

### Pirfenidone

Pirfenidone is an oral BS with antifibrotic and anti-inflammatory properties. Its only indication is the treatment of mild to moderate idiopathic pulmonary fibrosis. It exerts its effect by inhibiting transforming growth factor (TGF)-β1.

Skin rash was reported in 32% of patients treated with pirfenidone and in 12% of patients treated with placebo [27]. In addition, phototoxic burn-like skin rashes on sun-exposed body areas and erythematous (edematous or non-edematous) lesions were reported in 12% of patients and in 2% with placebo.

In newly published FDA labels, photosensitivity and rash were reported at a rate of 9%, but HSR and anaphylaxis were not mentioned in this report [28].

## Dermatology

Indications for which BSs are developed in dermatology include moderate to severe psoriasis, chronic urticaria, and atopic dermatitis (Table 3). Currently prescribed BSs include alefacept, efalizumab, ixekizumab, secukinumab, ustekinumab, dupilumab, quilizumab, ligelizumab, and omalizumab. TNF‑α inhibitors such as etanercept, infliximab, and adalimumab have also been approved by the FDA for treatment of moderate to severe psoriasis and psoriatic arthritis [29]. Off-label indications for TNF‑α inhibitors include autoimmune bullous disease, pemphigus vulgaris, and pyoderma gangrenosum [30]. Rarer indications include connective tissue disorders such as scleroderma, dermatomyositis, systemic lupus erythematosus, Sweet’s syndrome, sarcoidosis, granuloma annulare, toxic epidermal necrolysis, pityriasis rubra pilaris, and Behcet’s disease [29]. BSs used in the treatment of psoriatic arthritis will be mentioned in the section *Rheumatology*.

**Table 3:** Reported allergic reactions to biotechnological substances (Dermatology)

Biologics	ROA	Feature	Authors	Year	HSR %	IR %	ISR %	Urticaria %	Anaphylaxis %
Alefacept	i.m., i.v.	Human	FDA [31]	2012	0.2	–	16.0	<1.0	–
Efalizumab	s.c.	Humanized	Gordon et al. [32]	2003	–	–	–	–	0
			FDA [33]	2009	8.0		–	1.0	–
			Brunasso et al. [34]	2011	–		4.0	–	–
Ixekizumab	s.c.	Humanized	FDA [35]	2017	≤0.1	–	17.0	<0.1	–
			Strober et al. [36]	2017	0.1		6.8	<0.1	0
Secukinumab	s.c.	Human	EMA [37]	2015	6.5–11.2	–	5.6	<0.1	0
			Schwensen et al. [38]	2017	–		3.0	–	2.0
			FDA [39]	2018	–		–	0.6–1.2	–
			Deodhar et al. [40]	2019	2.4		0.8–1.3	–	–
Ustekinumab	i.v. s.c.	Human	EMA [41]	2017	–	–	3.0	–	0
			FDA [42]	2018	0.08	0.1	1.0–2.0	<0.1	<0.1
			Ghosh et al. [43]	2019	<1.0	0	–	<1.0	0
Dupilumab	s.c.	Human	FDA [44]	2017	<0.1	–	10.0	<1.0	–
			Ou et al. [45]	2018	–		13.2	–	–
			EMA [46]	2019	3.0–4.3		16.0–20.1	0.5–1.3	0.2
Ligelizumab	s.c	Humanized	Maurer et al. [47]	2019	–	–	4.0–7.0	–	0
Quilizumab	s.c.	Humanized	Harris et al. [48]	2016	–	–	6.9	–	–

*ROA* route of administration, *HSR* hypersensitivity reaction, *IR* infusion reaction, *ISR* Injection-site reaction, *i.m.* intramuscular, *s.c.* subcutaneous, *i.v.* intravenous, *FDA* Food and Drug Administration, *EMA* European Medicines Agency

### Alefacept

Alefacept is a fully human recombinant lymphocyte function-associated antigen-3 (LFA-3) immunoglobulin G1 fusion protein with a dual action mechanism that targets T cells, and can be administered intramuscularly or intravenously on a weekly basis. Its primary function is to interact with CD2 in the membrane of CD4 + and CD8 + T cells, inhibiting activation and thus regulating CD2/LFA‑3 interaction. A secondary mechanism of action is the induction of apoptosis in memory-effect T lymphocytes.

According to FDA labels, four out of 1869 patients (0.2%) reported angioedema in clinical trials: two of these patients were hospitalized and treated [31]. However, urticaria was seen in six patients (<1%) during the 24-week period. In one patient, therapy needed to be terminated due to allergic reactions. ISRs were reported in 16% of patients receiving alefacept by intramuscular administration, compared with 8% of patients treated with placebo. Repeated doses did not change the rate of incidence. ISRs were usually mild and were reported to manifest as pain (7%), inflammation (4%), bleeding (4%), edema (2%), local granuloma (1%), and nonspecific reaction or skin hypersensitivity (<1.0%). It has been reported that approximately 3% of patients developed low titer antibodies to the fusion protein, but a long-term effect was not known. FDA approval was withdrawn in September 2012 after a decision was made by the manufacturer to stop production of the drug, and the drug was never approved for the European market. There is no information about the frequency of anaphylaxis in the literature.

### Efalizumab

Efalizumab is a humanized mAb that binds to the CD11a subunit of lymphocyte function-associated antigen‑1 (LFA-1) on the surface of lymphocytes. It is used to treat adult patients with moderate to severe psoriasis.

In a randomized controlled study involving 556 patients conducted by Gordon et al., 2% of patients developed antibodies to efalizumab, but no anaphylaxis was observed [32].

In controlled clinical trials involving 1213 patients, 8% experienced at least one HSR (e.g., asthma, dyspnea, angioedema, urticaria, maculopapular rash) and 1% experienced urticaria. Other reported side effects include angioedema, laryngospasm, erythema multiforme, serum sickness-like reaction and allergic drug eruption. A low concentration of antibodies to protein components of the drug have been reported in 6.3% of patients (referenced in [33]).

In a retrospective cohort study conducted by Brunasso et al., ISRs (local erythema, itching, burning, pain, edema, or urticaria) were observed in 4% of participants [34].

### Ixekizumab

Ixekizumab is an immunosuppressive and anti-inflammatory mAb used to treat moderate to severe plaque psoriasis and can be administered subcutaneously. Its mechanism of action involves binding of the cytokine IL-17A, an important factor in keratinocyte activation and proliferation.

According to FDA labels, ISRs were reported in 17% of patients. The most common type of ISRs were erythema and pain [35]. Less frequently, serious HSRs such as angioedema and urticaria (≤0.1% each) have also occurred. In the post-marketing period, rare events of anaphylaxis requiring hospitalization have been reported.

In a long-term clinical investigation by Strober and colleagues [36] following 4209 patients, no anaphylactic reactions were reported. In this study, ISR, local erythema, and drug hypersensitivity were reported in 6.8%, 2.1%, and 0.1% of patient-years, respectively.

### Secukinumab

Secukinumab is a mAb that is administered subcutaneously to treat plaque psoriasis, ankylosing spondylitis, and psoriatic arthritis. Its anti-inflammatory and immunomodulatory properties result from its binding to and inactivation of IL-17A.

In the assessment report of the EMA in 2015, no cases of anaphylaxis or angioedema were reported [37]. Urticaria was rarely (<1.0%) observed. Injection-site pain was reported in 5.6% of cases, and general HSR occurred in 6.5–11.2%. The most common HSRs in any secukinumab group were reported to be dermatitis (1.2%), eczema (1.4%), and rash (1.8%). Less than 1% of patients developed antibodies during sekukinumab treatment for up to 52 weeks.

In one study, erythema and itching at the injection site were reported in 3.0% of participants and anaphylaxis in 2.0% [38]. According to the FDA label, urticaria was reported in 0.6–1.2% of patients depending on the dose, and less than 1% of patients developed ADA [39]. Although this report states that anaphylactic and allergic reactions are rarely seen, the rate was not given. In a recent study, HSR was reported to be 2.4% per 100 patient-years and ISR in 0.8–1.3%; in addition, ADAs were reported in <1% of patients receiving treatment for 52 weeks [40].

### Ustekinumab

Ustekinumab is a mAb that acts as an interleukin inhibitor and was approved for administration at 3‑month intervals as a second-line treatment for patients with moderate to severe plaque psoriasis. It has immunosuppressant and anti-inflammatory effects and neutralizes IL-12 and IL-23.

In the EMA evaluation report for 2017, ISRs were reported to occur in 3.0%. Intravenous infusion was not associated with anaphylaxis, IR, or serum sickness-like reactions [41].

FDA labels show a 1–2% incidence of ISRs (bruising, hemorrhage, induration, irritation, pain, pruritus, and swelling) [42]. In addition, one patient (0.1%) experienced signs and symptoms (flushing, tightness of the throat, shortness of breath) consistent with anaphylaxis after initial SC injection, and one patient (0.08%) experienced signs and symptoms consistent with or related to an HSR (urticaria, flushing, chest discomfort, and increased body temperature) after initial intravenous injection. In addition, approximately 6–12.4% of patients developed ADAs, which were generally of low titer (referenced in [42]).

A recent study examined the side effects of ustekinumab in psoriasis, psoriatic arthritis, and Crohn’s disease [43]. No serum sickness-like reactions or serious anaphylactic events were reported. Two patients (<1.0%) had temporary signs of treatment-associated hypersensitivity. The patients developed flushing, shortness of breath, and throat tightness after the first SC administration. In addition, fever, flushing, chest discomfort, and urticaria were observed after the initial intravenous administration.

Long-term safety and side effect studies are ongoing (C0168Z03 [PSOLAR] and CNTO1275PSO4005 [Nordic Database Initiative]).

### Dupilumab

Dupilumab is a relatively new BS in the mAb group, with anti-inflammatory and selective immunosuppressive properties. It is used as second-line therapy to treat moderate to severe atopic dermatitis. It exerts its effect by binding the alpha subunit of the IL‑4 receptor, eliminating the biological effects of IL‑4 and IL-13. Since May 2019, it has been additionally approved for severe uncontrolled asthma with type‑2 inflammation.

In the FDA drug report, hypersensitivity reactions such as serum sickness, serum sickness-like reaction, and generalized urticaria occurred with a frequency of <1%, and ISRs were observed in 10% of cases [44]. ADA were detected in 2–8% of treated patients, and two subjects developed serum sickness or serum sickness-like reactions. In these patients, a high antibody titer for dupilumab was reported during treatment. There is no information about anaphylaxis.

In a meta-analysis conducted by Ou et al., ISRs were reported in 13.2% of cases [45]. There is no mention of anaphylaxis, HSR, or urticaria, but it is stated that the high incidence of ISRs needs to be explained.

The EMA 2019 report shows an HSR in 3.0–4.3% of adult patients and ISRs in 16.0–20.1% (referenced in [46]). Severe ISRs were reported in 0.3–1.4%. Most ISRs were mild to moderate. The other most commonly reported adverse events were urticaria (0.5–1.3%) and rash (0.5–0.6%), depending on the dose, but with a similar incidence to placebo. Anaphylactic reaction or angioedema was reported in three patients (0.2%), hypersensitivity in two patients (0.1%), drug hypersensitivity in two patients (0.1%), and anaphylactic shock in one patient (<0.1%).

### Ligelizumab

Ligelizumab is a newly developed humanized mAb against human IgE and belongs to the IgG1/κ isotype subclass. This BS has 50-fold greater affinity to human IgE compared with omalizumab. A recent phase II clinical trial demonstrated that a higher percentage of patients had complete control of symptoms of chronic spontaneous urticaria with ligelizumab therapy of 72 mg or 240 mg than with omalizumab or placebo [47]. In the 72-mg treatment group in this study, mild injection site erythema was observed in 2.0% and mild to moderate ISRs in 4.0% of patients, while in the 240-mg-treated group, these were reported in 6.0% and 7.0%, respectively. In addition, serious side effects were reported in 2% of patients in both treatment groups, but no mortality or anaphylaxis occurred. Phase III studies are ongoing and expected to be finalized by 2021.

### Quilizumab

Quilizumab is a newly developed humanized mAb against the M1-prime segment of membrane-expressed IgE. It leads to depletion of IgE-producing and memory B cells, thus blocking IgE production. Its primary indications are chronic spontaneous urticaria and uncontrolled allergic asthma. In one study assessing efficacy and safety, 6.9% of patients reported ISRs (most often local pain) [48]. Further studies were discontinued due to the lack of efficacy in adults.

## Oncology

The use of BSs in oncology is quite extensive. The literature shows that these drugs are used for the treatment of bladder, breast, cervical, head and neck, gastrointestinal (stomach, bowel, colorectal), lung, and kidney cancers, as well as leukemia and lymphoma. Details of the reactions are given in Table 4.

**Table 4:** Reported allergic reactions to biotechnological substances (Oncology)

Biologics	ROA	Feature	Author	Year	HSR %	IR %	ISR %	Urticaria %	Anaphylaxis %
Aflibercept	i.v.	Human	EMA [49], FDA [50]	2019	0.3	–	–	–	–
Alemtuzumab	i.v. s.c.	Humanized	EMA [52]	2013	–	2.2–2.8	–	17.0	2.8
			BCCA [53]	2015	–	< 1.0	1.0	21.–30.0	<1.0
			FDA [54]	2019	–	92.0*	–	16.0	3.0
			Frau et al. [55]	2019	–	95.5*	–	–	–
Atezolizumab	i.v.	Humanized	FDA [56]	2016	–	1.3–1.7	–	–	–
			EMA [57]	2019	≤10.0	≤10.0		≥10.0	≤10.0
			BCCA [58]	2020	≤1.0	<1.0		8.–18.0**	≤1.0
Bevacizumab	i.v.	Humanized	BCCA [59]	2016	≤5.0	≤5.0	–	–	–
			EMA [60]	2019	2.5–5.9	0.3–6.1		–	2.5–5.9
			FDA [61]	2019	–	<3.0		–	–
Blinatumomab	i.v.	Murine	BCCA [62]	2017	2.0	29.0	–	–	–
			EMA [63]	2018	–	67.2		–	–
			FDA [64]	2019	–	77.0		–	–
Cetuximab	i.v.	Chimeric	Needle et al. [67]	2002	7.4	–	–	–	1.4
			Mariotte et al. [74]	2011	15.2	–		–	–
			Maggi et al. [65]	2011	–	1.2–15.0		–	–
			Galvão et al. [68]	2015	1.1–5.0	15.0–21.0		–	–
			FDA [69]	2019	–	8.4		–	–
Durvalumab	i.v.	Human	FDA [75]	2019	–	0.3–2.2	–	11.–26.0**	–
			EMA [76]	2020	–	0.3–1.9		1.6**	–
			BCCA [77]	2020	–	1.0–2.0		14.–26.0**	–
Gemtuzumab ozogamicin	i.v.	Humanized	FDA [78]	2006	0	8.0	22.0	–	–
			EMA [79]	2018	–	3.6–7.6	2.1–9.3	5.8–19.9**	–
Ibritumomab tiuxetan	i.v.	Mouse	FDA [81]	2002	2.0	–	–	4.0	<1.0
			EMA [82]	2017	<10.0	–	–	–	<1.0
			BCCA [83]	2017	<1.0	1.0	1.0–13.0	–	1.0–5.0
Ipilimumab	i.v.	Human	FDA [87]	2019	–	4.2–5.1	–	2.0	–
			EMA [88]	2020	<1.0	2.2–4.0		1.0–10.0	0.1–0.01
			BCCA [89]	2020	–	2.0–6.0		19.–26.0**	–
Necitumumab	i.v.	Human	FDA [91]	2015	–	1.5	–	–	–
			EMA [92]	2016	–	1.5		–	2.8
Nivolumab	i.v.	Human	FDA [93]	2019	–	6.4	–	–	–
			EMA [94]	2020	1.–10.0	1.–10.0		17.–65.0**	0.1–0.01
			BCCA [95]	2020	–	2.0–4.0		1.0	–
Panitumumab	i.v.	Human	FDA [100]	2015	–	1.0–4.0	–	–	–
			BCCA [101]	2020	1.0	3.0–4.0		–	1.0
Pembrolizumab	i.v.	Humanized	EMA [103]	2019	–	1.–10.0	–	1.5**	–
			BCCA [104]	2019	–	<1.0		–	–
			FDA [105]	2020	0.2	0.2		–	0.2
Pertuzumab	i.v.	Humanized	BCCA [107]	2014	11.0	13.–19.0	–	–	–
			EMA [108]	2019	1.–10.0	≥10.0		–	0.1–1.0
			FDA [109]	2020	5.–11.0	13.–21.0		–	5.–11.0
Ramucirumab	i.v.	Human	BCCA [110]	2017	–	1.–16.0	–	–	–
			FDA [111]	2019	–	1.–9.0		–	–
			EMA [112]	2019	–	<10.0		–	–
Trastuzumab	i.v. s.c.	Humanized	BCCA [113]	2020	3.0	21.0–40.0	–	–	–
			Galvão et al. [68]	2015	0.6–5.0	40.0	–	–	–
			EMA [114]	2018	0.9–3.5	8.5–37.1	–	–	0–0.9
			FDA [115]	2019	2.2–2.7	1.4–1.6	–	–	<1.0

*ROA* route of administration, *HSR* hypersensitivity reaction, *IR* infusion reaction, *ISR* Injection-site reaction, *s.c.* subcutaneous, *i.v.* intravenous, *FDA* Food and Drug Administration, *EMA* European Medicines Agency, *BCCA* British Columbia Cancer Agency

*mild to moderate or any grade of reaction when intravenously administered

**immune-mediated rash

### Aflibercept

Aflibercept is a human recombinant fusion protein targeting vascular endothelial growth factor 1 (VEGF-1) receptors used in colorectal carcinoma with metastasis, as well as retinal diseases such as macular degeneration.

In the EMA 2019 assessment report and FDA 2019 label, it was stated that HSRs were observed at a rate of 0.3% (placebo 0.5%) [49, 50]. Severe HSRs such as anaphylaxis, angioedema, bronchospasm, and dyspnea have been described as uncommon (≥1/1000 to <1/100) [49]. However, more detailed information has not been provided. In addition, ADA has been reported to develop in 1.4–3.1% of patients, and the clinical significance of their neutralization has not been assessed due to limited data [50]. Maculopapular rash may develop after intravitrael administration of this drug in retinal diseases [51].

### Alemtuzumab

Alemtuzumab is a monoclonal IgG1kappa antibody and a cytolytic agent used intravenously to treat leukemia and multiple sclerosis. This drug binds the glycoprotein CD52 on the surface of lymphocytes and other immune cells and leads to cell death.

In the 2013 EMA assessment report, allergic side effects were reported with relatively high frequencies (rash in 48%, urticaria in 17%, and pruritus in 16.5% of patients) [52]. Serious and severe urticaria was seen in 0.4% of cases. Severe infusion-associated reactions, including pyrexia, urticaria, atrial fibrillation, nausea, chest discomfort, hypotension, and grade IV anaphylaxis were reported in 2.2–2.8% of cases.

The British Columbia Cancer Agency (BCCA) Drug Manual reported anaphylactic reactions and angioedema in <1%, ISRs in 1.0% (severe in 0%) of intravenous users and ISRs in 90% (severe in 2%) of SC users. Reactions occurred between 24 h after the first dose and the administration of the fourth dose. In addition, SC injection site pain was reported in 61.0% (severe in 2.0%). Pruritus and urticaria were reported in 21–30% (1–5% of these reactions were severe; 0% of the reactions was associated with SC use) (referenced in [53]).

In the last published FDA label, IRs and cytokine release syndrome were reported in 92% of treated patients, but epinephrine or atropine was administered in 0.6% of these patients [54]. In some patients, infusion reactions occurred 24 h after infusion. Symptoms include fatigue, dyspepsia, pruritus, flushing, urticaria, dyspnea, pain, nausea, dizziness, and pulmonary infiltrates. Severe reactions occurred in approximately 3% of patients. Two patients experienced anaphylaxis (including anaphylactic shock). Symptoms include headache, rash, chest pain, angioedema, bradycardia, tachycardia (including atrial fibrillation), bronchospasm, hypotension, hypertension, transient neurological symptoms, and pyrexia. The most common side effects were rash (53%), urticaria (16%), pruritus (14%), and flushing (10%). Anti-alemtuzumab antibodies were detected in 62%, 67%, 29%, and 75% of the patients at 1, 3, 12, and 24 months of treatment, respectively.

In a recent study that evaluated efficacy and safety, 95.5% of patients had IRs such as fever, shivers, rash, and headache [55].

### Atezolizumab

Atezolizumab is an IgG‑1 class humanized antibody that acts by binding to programmed death ligand 1 (PD-L1) used to treat bladder, breast, and lung cancer. PD-L1 is the immune checkpoint protein, which is expressed by tumor cells and inhibits the anti-tumor function of the T cell. Inhibition of the ligand’s interaction with its receptor by BS enhances the anti-tumoral activity of T cells and improves the immune system of the patients.

According to the FDA 2016 label, severe IRs were observed in 1.3–1.7% of patients [56]. These reactions include the following symptoms: back or neck pain, chills or shaking, dizziness, feeling like passing out, fever, flushing, itching or rash, dyspnea or wheezing, and swelling of face or lips. However, immune-related adverse reactions (such as colitis, hepatitis, hypophysitis, meningoencephalitis, pancreatitis, and pneumonitis) have been reported that affect various organs.

In the EMA 2019 assessment report, HSR such as anaphylaxis and IRs is reported to be seen in up to 10% of patients [57]. In addition, itching of the skin and rash are reported to be seen in more than 10% of patients.

According to the recent BCCA Drug Manual, it was reported that HSR including anaphylaxis can develop in ≤1% (severe <1%), IRs in 1% (severe ≤1%), and immune-mediated rash in 8–18% (severe ≤1%) of patients [58].

### Bevacizumab

Bevacizumab is a mAb used to treat various cancers including kidney, stomach, colon, breast, and lung cancer. The antibody inhibits binding of VEGF to its receptors, reducing angiogenesis (blood vessel formation) and tumor growth.

According to the BCCA Drug Manual, IRs/HSRs with symptoms (dyspnea, redness, rash, hypo- or hypertension, oxygen desaturation, chest pain, tremor, and nausea/vomiting) were reported in up to 5% of patients (referenced in [59]). Serious hypersensitivity reactions involving anaphylactic and anaphylactoid responses have occurred, but no rates have been reported. In addition, rash was reported in 13%, rhinorrhoea in 4%, and flushing in 1% of patients.

In the EMA assessment report, IRs occurred in 0.3–6.1% of cases, and the most commonly reported IR was hypertension (2.8–3.1%) (referenced in [60]). In this report, HSRs and anaphylaxis were described as treatment-emergent adverse events (TEAE) and were reported to be any grade of TEAEs in 36.9%, grade 3 or higher TEAEs in 5.9%, and serious TEAEs in 2.5% of cases. Further information on reactions is not available.

IR and symptoms (chest pain, chills, grade 3 hypersensitivity, diaphoresis, headache, hypertensive crises associated with hypertension, hypoxia, neurological signs and symptoms, and wheezing) have been reported in clinical trials and post-marketing studies, as indicated on the FDA label [61]. Any grade of IRs are reported to be rare (<3%), and serious IRs have occurred in 0.2% of the patients. Symptoms include chest pain, diaphoresis, hypertensive crises, hypoxia, rigors, and wheezing. Although urticaria and anaphylaxis are not mentioned in this label, exfoliative rash was seen in 23% with the administration of this BS in combination with the chemotherapy protocol.

### Blinatumomab

Blinatumomab is a bispecific mAb used for cancer immunotherapy and specific forms of acute lymphoblastic leukemia (ALL). It exerts its effect in part by simultaneously binding CD19 on B cells and CD3 on T cells.

This BS can lead to cytokine release syndrome (CRS) in 11–12% of patients, with 1% of these cases being severe, to hypersensitivity in 2% of patients, and infusion reactions in 29% of patients, according to the BCCA’s 2017 report (referenced in [62]). Symptoms of this syndrome include asthenia, nausea, pyrexia, headache, hypotension, and increased liver enzymes. It was reported that CRS peaked within the first 2 days of treatment and was not easily differentiated from IR. Although anaphylaxis and urticaria were not mentioned in this report, the incidence of rash was reported to be 12–21% (severe 2–3%).

According to the EMA assessment report, CRS occurred in 0.5–11.4% of patients, rash occurred in 4.3%, and IRs occurred in 67.2% (grade ≥3 in 14.3%). In total, severe IRs were very rarely seen (4.3%) [63]. Fatal IRs were not reported. Additionally, maculopapular rash and flushing have been reported in 4.3% of patients.

According to the recent FDA label, this BS caused CRS in 7–15% of patients (3% of these instances were severe), skin rash in 16% of patients (<1% of these instances were severe) and IRs in 77% of patients (5% of these instances were severe) [64]. Symptoms of CRS included asthenia, headache, nausea, fever, hypotension, increased liver enzymes, increased total bilirubin, and diffuse intravascular coagulation. IRs usually occurred during the first 48 h of infusion and lasted less than 2 days. Clinical findings included hypotension, hypertension, rash, generalized pruritus, pyrexia, periorbital edema, and cytokine release syndrome. Events defined as rash included maculopapular rash, erythema, contact dermatitis, and eczema.

### Cetuximab

Cetuximab is an intravenously administered epidermal growth factor receptor (EGFR) inhibitor used to treat advanced colorectal cancer, head and neck squamous cell carcinoma, pancreatic cancer, and less frequently, non-small cell lung cancer. This antibody can reduce cancer cell survival, neovascularization, cell migration, and metastasis. Cetuximab was the first anti-EGFR mAb and BS approved for cancer treatment; therefore, its immunological and allergic side effects are well studied.

In a review of acute infusion reactions induced by mAbs, acute IRs with cetuximab were in the range of 1.2–15% (referenced in [65]). It is noted in that particular review that the pathogenic mechanism of mAb-related IRs has not yet been fully defined, with the exception of several suggested mechanisms by W. J. Pichler [66].

HSRs have been reported in 7.4% of participants in a safety study with 419 patients, with 2.6% of reported HSRs being of grade 3 severity, and 1.4% being of grade 4 anaphylactic reactions [67]. The majority of reactions occurred after the first dose, and all grade 4 reactions occurred within minutes following the first infusion.

A 2015 review reported HSR to be in the 1.1–5.0% range, and infusion reactions in the 15.0–21.0% range in patients treated with cetuximab (referenced in [68]).

According to the FDA label, mild IRs (fever, chills, shortness of breath, bronchospasm, angioedema, urticaria, hypertension, and hypotension) occurred in 8.4% of patients, and severe IRs occurred in 2.2% of patients, with 90% of events occurring after the first infusion [69]. In addition, 82% of patients developed acneiform rash during treatment, 9.7% of which were severe. Anaphylactic reactions during treatment have been reported to be increased in association with tick bites by *Amblyomma americanum*, a history of red meat allergy, all associated with IgE-antibodies against α‑gal [69–71].

In 2010, reactions during the first infusion of cetuximab and delayed type anaphylactic reactions to red meat were investigated in detail [70]. In both diseases, patients were shown to have increased IgE antibodies specific for α‑gal, a mammalian disaccharide, not expressed in humans but on the tissue of mammalians. In most patients with an HSR and anaphylaxis, IgE antibodies to cetuximab were also present in serum before treatment, which prompted the investigations into the sensitization route [71]. It was shown that α‑gal is also the main cause of delayed anaphylaxis against red meat and that this was associated with the occurrence of the American tick *Amblyomma americanum *[72]. Presently, tick bites are thought to be the predominant sensitization route for anti-α-GAL IgE induction.

α‑GAL, however, has another implication for drug-induced hypersensitivity reactions: It is present in mammalian gelatine and, therefore, may be part of vaccines, gelatine-containing infusion solutions, ovula, capsules and pills, suppositories, snake venom antisera, and animal-derived heart valves (referenced in [73]).

However, in a study involving 92 patients, 14 (15.2%) reported HSR, eight of these (57.1%) reported severe HSR [74]. Anti-cetuximab IgE levels were detected in seven of the eight patients with severe HSR.

### Durvalumab

Durvalumab is a PD-L1 binding and blocking mAb used in non-small cell lung carcinoma and metastatic urothelial carcinoma.

In the FDA 2019 labels, IRs were reported to occur in 2.2% (grade 3 in 0.3%), and immune-mediated rash in 11–26.0% (grade 3–4 in 0.6–1.0%) of patients [75]. ADA developed in 2.9% of patients, but its clinical significance has not been established. HSRs and anaphylaxis are not mentioned in these labels.

In the EMA 2020 assessment report, any grade of IR has been reported to occur in 1.9% (grade 3 in 0.3%), and skin rash in 21.7% (grade 3 in 0.6%) of patients [76]. Symptoms of IR include: chills or shaking, dizziness, dyspnea or wheezing, fever, flushing, and itching or rash. HSRs and anaphylaxis are not mentioned in these labels.

According to the BCCA’s 2020 drug report, IRs can be observed in 1.0–2.0% (severe in <1%) of patients, and skin rash in 14–26.0% (severe in <1%) of patients [77].

### Gemtuzumab ozogamicin

Gemtuzumab (-ozogamicin) is an antibody against glycoprotein CD33, which is administered intravenously to treat acute myeloid leukemia (AML). This antibody combined with a cell-toxic substance induces apoptosis of the targeted cancer cells.

In a prospective observational study involving 225 patients, as described in the FDA report, IRs were observed in 8.0% of patients, and fatal reactions in 0.4% [78]. In addition, local reactions to the application occurred in 22.0%. Pruritus was described in 6.0% and rash in 18.0% of patients. Infusion-related reactions usually developed within 24 h after infusion. Signs and symptoms were similar to anaphylactic reactions. These included chills, hypoxia, fever, tachycardia, bronchospasm, and respiratory failure.

According to the 2018 EMA assessment report, IRs, including anaphylaxis, were reported in 7.6% of patients, and severe reactions in 3.6% of patients [79]. Symptoms included fever, chills, injection-site urticaria, and less frequently, hypotension, tachycardia, and respiratory symptoms. All symptoms occurred during the first 24 h after drug administration. In addition, rash was reported in 19.9% (severe in 5.8%), local erythema in 9.3% (severe in 2.1%), and pruritus in 5.4% (severe in 0.3%) of patients.

The literature contains one case report of HSR mortality, which was associated with platelet transfusion after gemtuzumab treatment [80].

### Ibritumomab tiuxetan

Ibritumomab (-tiuxetan) is used in non-Hodgkin’s lymphoma patients or for cancers that are refractory to rituximab. It acts by binding to the antigen CD20, which is present on the surface of malignant and normal B‑lymphocytes.

In the first FDA report, allergic reactions were reported in 2% of patients, urticaria in 4%, and severe life-threatening reactions such as angioedema, lung edema, tachycardia, subdural hematoma, and pulmonary embolus in <1% of patients [81].

In the EMA evaluation report, allergic (hypersensitivity) reactions and infusion reactions were described [82]. Common symptoms (up to one in 10 patients) include skin reactions, difficulty in breathing, swelling, itching, redness, chills, and dizziness (a potential marker for hypotension). Severe hypersensitivity reactions involving anaphylaxis occurred in <1% of patients.

According to the BCCA’s drug report, ISRs (dermatitis, desquamation, and ulcer following extravasation) occurred in 1.0% of patients, angioedema in 5.0% (severe angioedema in 1.0%), pruritus in 9.0%, rash in 8.0%, and mucocutaneous reactions in <1% of patients (referenced in [83]). That particular monograph states that rituximab is an essential component of ibritumomab-based treatment regimens. Infusion reactions may occur frequently after pretreatment with rituximab, or during or after ibritumomab administration. Asthenia, cough, dizziness, nausea, pruritus, pyrexia, rash, rigors, tachycardia, and vomiting have been reported among the symptoms of IRs.

In a recent case report, patients were shown to develop human anti-murine antibodies (HAMA) after exposure to murine antibodies [84]. The low incidence of hypersensitivity reactions in patients with high HAMA titers is particularly promising for ibritumomab.

### Imatinib

Imatinib is an active ingredient in capsules or tablets containing a group of kinase inhibitors used to treat chronic myeloid leukemia. Other and rarer indications include acute lymphoblastic leukemia, skin tumors, some gastrointestinal tumors, hypereosinophilic syndrome, atypical myelodysplastic or myeloproliferative disorders, as well as systemic mastocytosis.

The first FDA report does not include information on hypersensitivity and anaphylactic reactions. This report includes only skin rash in 32–39% of patients (severe skin rash in 3–4%), and pruritus in 6–10% (severe in 0.4–1.0%) [85].

BCCA drug manuals describe cutaneous reactions in <1%, rash in 32–39% (severe rash in 3–4%), and pruritus in 6–10% (with severe pruritus in 0–1%) of patients. This manual does not include information on hypersensitivity and anaphylactic reactions [86].

### Ipilimumab

Ipilimumab is a fully-human mAb used primarily for the treatment of metastatic melanoma, metastatic colorectal carcinoma, and renal cell carcinoma. This BS shows its effect by binding and inhibiting human cytotoxic T lymphocyte antigen 4 (CTLA-4). As a result, T cell infiltration increases in tumor cells and tissues, thereby leading to their destruction. In addition, studies are ongoing for its use in the treatment of lung cancer, bladder cancer, and metastatic prostate cancer.

In the FDA 2019 labels, IRs were reported to occur in 4.2–5.1% of patients and urticaria in 2.0% of patients [87]. Symptoms of IR include: chills or shaking, dizziness, dyspnea, feeling like passing out, fever, flushing, and itching or rash.

In the EMA 2020 assessment report, HSRs are reported as uncommon (≥1/1000–<1/100), urticaria as common (≥1/100 to <1/10), and anaphylactic reaction as very rare (<1/10,000) [88]. IRs were observed at a rate of 2.2–4.0% depending on the dose of the drug, but grade 3–5 IRs were not reported.

According to the BCCA’s 2020 drug report, IRs can be observed in 2.0–6.0% (severe in <1%) of patients, pruritus in 24–26.0% of patients, and skin rash in 19–26.0% (severe in <1%) of patients [89]. Additionally, a case that developed a delayed type of HSR during melanoma treatment has been reported [90].

### Necitumumab

Necitumumab is a human mAb used in chemotherapy combinations in the treatment of non-small cell lung cancer, and shows its effect by binding to the EGFR. EGFR plays an important role in the uncontrolled growth and proliferation of tumor cells and tissue as a result of apoptosis inhibition.

In the FDA 2015 labels, IRs of any severity have been reported to occur in 1.5% (grade 3 in 0.4%) of patients [91]. These reactions have been frequently observed after the first two doses.

The same data are also reported in the EMA 2016 assessment report [92]. In addition, it is stated that skin reactions can be seen in 77.9% (grade 3 in 6.3%) of patients. Symptoms of IR include: chills, dyspnea, or fever. Urticaria and anaphylaxis are not mentioned in that report. In addition, sudden death or cardiorespiratory arrest was reported in 2.8% of patients, which may also be a symptom of anaphylaxis.

### Nivolumab

Nivolumab is a human mAb that is used in the treatment of advanced renal cell carcinoma, hepatocellular carcinoma, Hodgkin’s lymphoma, melanoma, non-small cell lung cancer, squamous cell cancer of the head and neck, as well as urothelial cancer and acts by binding to the programmed cell death protein (PD)-1-receptor.

In the FDA 2019 labels, IRs were reported to occur in 6.4% of patients [93]. The IR causing permanent discontinuation or withholding of the BS was reported in 0.5–1.4% of patients within 48 h after infusion. Anaphylaxis and hypersensitivity have not been reported, but it has been reported that the incidence of symptoms such as abdominal pain, arthralgia, asthenia, back pain, constipation, cough, decreased appetite, diarrhea, dyspnea, fatigue, headache, musculoskeletal pain, nausea, pruritus, pyrexia, rash, upper respiratory tract infection, and vomiting is more than 20%. Some of these symptoms can be seen in anaphylaxis. In treatment with this BS, immune-mediated organopathies such as colitis, encephalitis, hepatitis, pneumonitis, endocrinopathies, and nephritis can also be observed. The clinical significance of ADAs for these side effects is still unknown.

In the EMA 2020 assessment report, IRs and HSRs are stated as common (≥1/100–<1/10), rash and prutitus as very common (≥1/10), and anaphylactic reaction as rare (≥1/10,000–<1/1000) [94].

According to the BCCA’s 2020 drug report, IRs can be observed in 2.0–4.0% of patients, pruritus in 7–17.0% of patients, skin rash in 11–21.0% (severe in 1.0%), and urticaria in 1.0% of patients [95].

Allergic symptoms (eyelid angioedema, flushing, and hives on the neck and face) were reported in one patient during treatment with nivolumab [96]. However, a case with cytokine release syndrome has been reported [97]. In a recent case report, a patient who had recurrent infusion reactions despite premedication against nivolumab was successfully treated with pembrolizumab, another PD-L1/PD-L2-inhibitor [98].

### Panitumumab

Panitumumab is a mAb EGFR inhibitor delivered intravenously to treat metastatic colorectal cancer. It acts by binding and inhibiting EGFR. Panitumab appears to be a safe option for treatment and potential side effects in patients with anti-α-gal IgE antibodies [99]. This is due to the fact that α‑galactosylated glycans are absent in purely human mAbs and that the system in which it is expressed does not seem to induce posttranslational modifications like allergenic glycosylation. The chimeric mAb cetuximab is not suitable in this sensitive patient group due to its high α‑gal content.

In the 2015 FDA report, IRs were reported in 4% of patients (severe reactions in 1%) [100]. Severe IRs included anaphylactic reactions, bronchospasm, and hypotension. Angioedema and fatal reactions have also been documented in post-marketing reports.

In the BCCA drug monograph, HSR was reported in 1% of patients (occurring within 24 h), while IRs were reported in 3–4% (severe IRs were seen in 1%, occurring within 24 h) (referenced in [101]). Angioedema was seen very rarely. Some cases were fatal, with possible late onset more than 24 h post infusion. The symptoms of most IRs were mild and included chills, fever, or dyspnea. In addition, pruritus was reported in 34–69% (severe in 1–4%) and rash in 20–78% (severe in 1–3%).

At the annual congress of the European Academy of Allergy and Clinical Immunology (EAACI) in 2019, a case of a successful desensitization was presented in a patient that had developed anaphylaxis against panitumumab [102].

### Pembrolizumab

Pembrolizumab is a monoclonal antibody that is used to treat non-small cell lung cancer, lymphoma, melanoma, and urothelial carcinoma and which binds to the PD-1-receptor and inhibits its interaction with its ligands.

In the EMA 2019 assessment report, IRs are stated as common (≥1/100–<1/10), rash and pruritus as very common (≥1/10), and gastrointestinal symptoms (such as abdominal pain, constipation, diarrhea, nausea, vomiting) as very common (≥1/10) [103]. According to the BCCA’s 2019 drug report, IRs can be observed in <1.0% of patients, pruritus in 12–30.0% of patients, and skin rash in 1.0–29.0% (severe in 1.0%) of patients [104].

In the FDA 2020 labels, severe or life-threatening anaphylaxis, HSRs or IRs were reported in 0.2% of patients [105]. Symptoms of IR include: chills, dizziness, fever, flushing, hypotension, hypoxemia, pruritus, rash, rigors, and wheezing. However, the incidence of ADAs against pembrolizumab was 1.8%, and it was reported that ADA had no clinical relevance [106].

### Pertuzumab

Pertuzumab is a humanized mAb that acts by blocking the human epidermal growth factor receptor 2 protein (HER2), and is used in combination with trastuzumab and docetaxel in the treatment of breast cancer. HER2 plays an important role in the growth and differentiation of tumor cells, and its blocking results in apoptosis.

According to the BCCA’s 2014 drug report, HSRs can be observed in 11.0% (severe in 2–5%) of patients, IRs in 13–19.0%, rash in 10–20% (severe in 2%), and pruritus in 10% of patients [107]. Symptoms of IR include: asthenia, chills, fatigue, fever, hypersensitivity, and vomiting.

In the EMA 2020 assessment report, IRs and rash are stated as very common (≥1/10), HSRs as common (≥1/100 to <1/10), anaphylaxis as uncommon (≥1/1000–<1/100), and CRS as rare (≥1/10,000–<1/1000) [108]. The most common IRs have been reported as asthenia, chills, fatigue, headache, hypersensitivity, pyrexia, and vomiting.

In the FDA 2019 labels, IRs have been reported to occur in 13–21.0% (severe less than 1.0%), and hypersensitivity/anaphylaxis in 5.0–11.0% of patients [109]. It is stated that CRS may also develop.

### Ramucirumab

Ramucirumab is a human mAb used in the treatment of gastric, breast, lung, and colorectal cancers, showing its effect by selectively inhibiting VEGF receptor‑2. VEGF plays an important role in angiogenesis, cell division, and migration in vascular endothelium.

According to the BCCA’s 2017 drug report, IRs can be observed in 1.0–16.0%, and rash in 4.0% of patients [110]. Symptoms of IR include: back pain, chest pain, chills, dyspnea, flushing, paresthesias, rigors, and wheezing. Some cases have been observed with more severe symptoms such as bronchospasm, hypotension, and supraventricular tachycardia.

In the FDA 2019 labels, IRs have been reported to occur in 1.0–9.0% (severe in <1.0%) of patients [111]. Symptoms of IR include: back pain/spasms, chills, chest pain and/or tightness, dyspnea, flushing, hypoxia, paresthesia, rigors/tremors, and wheezing.

In the EMA 2019 assessment report, IRs can be observed in up to 10.0% of patients [112]. Symptoms of IR include: back or chest pain, chest tightness, chills, dyspnea, feeling of tingling or numbness in hands or feet, flushing, increased muscle tension, and wheezing. Some cases have been observed with more severe symptoms such as breathing distress, feeling faint, and tachycardia. Anaphylaxis and hypersensitivity are not mentioned in that report.

### Trastuzumab

Trastuzumab is an anti-HER2-antibody combined with or conjugated to a cytotoxic drug that is used to treat local or advanced inoperable breast and gastric cancer.

In the BCCA drug assessment report (referenced in [113]), IRs occurred in 21–40% of cases, with severe reactions in 1%. Allergic reactions were reported in 3% of cases. IRs occurred in 40% of patients with their first infusion. Mild to moderate symptoms included headache, dizziness, rash, cough, nausea, vomiting, pain, chills, and asthenia. Severe reactions and symptoms were most often respiratory distress, bronchospasm, wheezing, low oxygen saturation, and hypo- or hypertension. Galvão et al. (referenced in [68]) reported an HSR rate of 0.6–5.0% and an infusion-related reaction rate of 40%.

According to the EMA assessment report 2018, HSR was reported in 0.9–3.5%, IRs in 8.5–37.1%, and anaphylaxis in 0–0.9% of patients [114].

According to the FDA’s 2019 report, IRs occur in 1.4–1.6% of patients and include symptoms such as fever, chills, redness, shortness of breath, bronchospasm, wheezing, tachycardia, and hypotension. In the same report, pruritus was seen in 6.0% and drug hypersensitivity was found to occur in 2.2–2.7% of patients. In preliminary studies, anaphylaxis was seen in one patient [115].

## Rheumatology

BSs are often used to treat rheumatoid arthritis, psoriatic arthritis, ankylosing spondylitis, inflammatory bowel diseases (ulcerative colitis, Crohn’s disease), Still’s disease, and vasculitis in rheumatology. Details on the reactions are given in Table 5.

**Table 5:** Reported allergic reactions to biotechnological substances (Rheumatology)

Biologics	ROA	Feature	Author	Year	HSR %	IR %	ISR %	Urticaria %	Anaphylaxis %
Adalimumab	s.c.	Human	Puxeddu et al. [116]	2012	3.5	–	1.5	1.5	0
			Tarkiainen et al. [117]	2015	18.1		17.0	–	–
			FDA [118]	2018	6.0		8.0–20.0	6.0**	–
Anakinra	s.c.	Human	EMA [119]	2014	–	–	≥10.0	0.1–1.0	0.1–1.0
			FDA [120]	2018	–		71.0*	–	–
Belimumab	*s.c.* *i.v.*	*Human*	EMA [124]	2018	< 10.0	7.0–12.0	6.1	–	0.1–1.0
			FDA [125]	2019	13.0	17.0	–	–	0.6
Canakinumab	*s.c.*	*Human*	FDA [126]	2016	–	–	≥10.0	–	0
Etanercept	s.c.	Humanized	Puxeddu et al. [116]	2012	5.3	–	1.6	2.0	0.8
			Tarkiainen et al. [117]	2015	11.3		7.5	–	–
			FDA [128]	2018	<2.0		15–37.0	2.0	<2.0
Golimumab	s.c.	Human	Kay et al. [129]	2015	–	–	4.7–11.6	–	0
			FDA [130]	2018	–		3.4–6.0	–	0
Infliximab	i.v.	Chimeric	Maggi et al. [65]	2011	–	1.0–27.0	–	–	–
			Puxeddu et al. [116]	2012	13.8	–	0	4.4	9.3
			Tarkiainen et al. [117]	2015	34.1	–	1.9	–	1.9
			FDA [131]	2019	–	18.0	–	<1.0	<1.0
Certolizumab	s.c.	Humanized	EMA [134]	2015	0.2–1.1	–	6.4	–	–
			FDA [135]	2019	–		1.7–3.2	0.3	–
Rituximab	i.v. s.c.	Chimeric	Maggi et al. [65]	2011	–	10–77.0	–	–	–
			FDA (s.c.) [137]	2017	–	–	16–26.0	–	–
			FDA (i.v.) [138]	2018	–	≥25.0	–	2.0–8.0	–
			BCCA [139]	2018	1–10.0	14–77.0	20.0	7.0	–

*ROA* route of administration, *HSR* hypersensitivity reaction, *IR* infusion reaction, *ISR* Injection-site reaction, *s.c.* subcutaneous, *i.v.* intravenous, *FDA* Food and Drug Administration, *EMA* European Medicines Agency, *BCCA* British Columbia Cancer Agency

*any grade of reactions

**immune-mediated rash

### Adalimumab

Adalimumab is a recombinant human IgG1 that binds to TNF‑α with high affinity. It acts by inhibiting the binding of TNF‑α to p55 and p75 surface receptors on target cells.

In a study on 201 patients conducted by Puxeddu et al. [116], hypersensitivity reactions occurred in seven patients (3.5%), local reactions occurred in three patients (1.5%), and urticaria and angioedema occurred in three patients (1.5%). Anaphylaxis was not observed in this study, but has been reported very rarely in the post-marketing period. Another study conducted by Tarkiainen et al. [117] reported HSR frequencies of 18.1%, ISRs frequencies of 17.0%, and allergic symptoms in 1.1% of the patients.

FDA 2018 labeling showed that local ISRs are more common in patients treated with the TNF‑α inhibitor than in those treated with placebo [118]. The incidence of ISRs including erythema, itching, hemorrhage, and pain or swelling is 8.0–20.0% in patients treated with adalimumab vs 1% in patients treated with placebo. Anaphylaxis and angioneurotic edema have rarely been reported following the application of this BS, and in the first 48 weeks of treatment, approximately 6% of patients had non-severe local allergic hypersensitivity reactions and allergic rashes.

### Anakinra

Anakinra is a genetically engineered and recombinant human IL‑1 receptor antagonist. Its main effect is by neutralizing the biological activity of IL-1α and IL-1β, a proinflammatory cytokine. As a result, it contributes to the reduction of synovial inflammation by reducing many cellular responses.

However, anakinra has the following therapeutic indications: rheumatoid arthritis (RA), cryopyrin-associated periodic syndrome (CAPS), and Still’s disease.

In the EMA summary of product characteristics (SmPC) report, ISRs are generally reported to occur in the first 2 weeks of treatment (for RA and Still patients) and resolve within 4–6 weeks [119]. In that particular report, allergic reactions such as anaphylactic reactions, angioedema, urticaria and pruritus are expected to range from 1/100 to 1/1000.

According to the FDA’s 2018 label, the most frequently reported adverse reactions were ISRs (71.0%), which were mild to moderate in the majority of patients [120]. Only 3.2% of these reactions were classified as severe, with the majority including ecchymosis, erythema, inflammation, and local pain. Reactions usually occurred during the first month. This label states that anaphylaxis, angioedema, urticaria, and rash may occur, but no specific rate or detailed information is provided.

In the literature, it has been reported that anakinra treatment may cause anaphylaxis, cutaneous drug reactions, erythematous plaques, pruritic rash, and severe HSRs [121]. In a pediatric case, a patient with anakinra-induced anaphylaxis was successfully treated with canakinumab, an alternative anti-IL‑1 agent [122]. However, a desensitization protocol is also available [123].

### Belimumab

Belimumab is a recombinant human IgG1 mAb that binds and inhibits the biological activity of the soluble B lymphocyte stimulator (BLyS), a member of the TNF ligand superfamily. It is used in the treatment of systemic lupus erythematosus and is applied subcutaneously or intravenously.

In the EMA 2018 assessment report, any event of ISRs (local pain, erythema, hematoma, induration, and pruritus) were reported in 6.1% of patients, HSRs in < 10.0% of patients, and post-injection anaphylactic reactions were reported in 0.1–1.0% of patients (serious reactions, 0.9%) [124]. HSR and anaphylactic reaction are mentioned to occur mostly during the first two infusions.

In the newly published FDA report, intravenous administration was evaluated [125]. HSRs were reported in 13.0% (in the placebo group 11%), anaphylaxis in 0.6% (placebo 0.4%) of patients. Symptoms included angioedema, dyspnea, hypotension, pruritus, rash, or urticaria. IRs were observed in 17% of patients and severe IRs in 0.5%, with placebo in 15% and 0.4%, respectively. It is stated that it is not easy to differentiate between HSRs and IRs due to the overlap in signs and symptoms. However, it has been reported that there is insufficient evidence as to how the premedication of some patients affects the frequency or severity of reactions.

### Canakinumab

Canakinumab is a human mAb against IL‑1 beta and indicated in periodic fever syndromes, CAPS, tumor necrosis factor receptor-associated periodic syndrome (TRAPS), familial Mediterranean fever (FMF), Still’s disease, and gouty arthritis.

According to the FDA’s 2016 label, the most frequent adverse drug reactions were infections predominantly of the upper respiratory tract, and mild to moderate ISRs equal or greater than 10.0% [126]. No anaphylactoid or anaphylactic reactions were observed in more than 2600 patients whose clinical development was tested for this biologic, but the risk of severe hypersensitivity reactions was mentioned.

No cases of canakinumab-related anaphylaxis have been reported in the current literature [127]. In addition, patients with anaphylaxis to anakinra have been shown to tolerate canakinumab very well [122].

### Etanercept

Etanercept is a subcutaneously administered selective immunosuppressive and anti-inflammatory BS belonging to the group of TNF‑α inhibitors used to treat rheumatoid arthritis and other rheumatic diseases. It exerts its effect by inhibiting the activity of TNF‑α.

Puxeddu et al. reported a 5.3% rate of HSRs, a 0.8% rate of anaphylaxis, a 2.0% rate of angioedema or urticaria, and a 1.6% rate of local reactions in a study involving 245 patients treated with etanercept [116]. In another study, the rate of HSRs was 11.3%, that of ISRs was 7.5%, and reactions at sites other than the injection site occurred at a rate of 4.2% [117].

The FDA drug label published in 2018 reports that allergic reactions associated with this treatment occur in <2% of patients, and mild to moderate ISRs (erythema, itching, pain, swelling, bleeding, bruising) occur in 15–37% of patients during the first 3 months of treatment [128]. ISRs were usually seen in the first month, and with decreased frequency thereafter.

### Golimumab

Golimumab is a selective immunosuppressive and anti-inflammatory TNF-α-inhibitor applied subcutaneously to treat rheumatoid and psoriatic arthritis, as well as ankylosing spondylitis. It is a mAb that inhibits the activity of soluble and membrane-bound forms of the proinflammatory cytokine TNF‑α by binding TNF‑α and inhibiting its receptor binding.

In a 3-year safety study, the frequency of ISRs was 4.7–11.6%, depending on the drug dose. No anaphylactic reactions or serum-like reactions have been reported to date during the follow-up period of up to 160 weeks [129].

The FDA 2018 drug label reports an ISR frequency of 3.4–6.0% [130]. The reactions were often mild and included erythema, urticaria, induration, and pain. No anaphylactic reactions were observed in phase II and III studies with the drug.

### Infliximab

Infliximab is an intravenously administered selective immunosuppressive and anti-inflammatory TNF‑α inhibitor used to treat rheumatoid and psoriatic arthritis, Crohn’s disease, and ankylosing spondylitis. This BS is a human–murine chimeric monoclonal IgG1κ antibody that binds TNF‑α and inhibits its effects.

In a review by Maggi et al. summarizing nine studies, infusion reactions occurred in 1–27% of patients depending on the disease being treated (referenced in [65]). In a study on 225 patients, Puxeddu and colleagues reported hypersensitivity reactions in 13.8%, anaphylaxis in 9.3%, urticaria or angioedema in 4.4%, and local reactions in 0% of patients [116]. Another study reported a 34.1% rate of infusion-related HSRs, and a 1.9% rate of anaphylaxis [117].

The FDA 2019 drug label reports IRs in 18% of patients during or within 1 h after the infusion [131]. A total of 3% of all patients given infliximab infusions experienced nonspecific symptoms (fever or tremor), while 1% experienced cardiopulmonary reactions (chest pain, hypotension, hypertension, or dyspnea). Symptoms related to pruritus, urticaria, and cardiopulmonary reactions were detected in 1% of patients. Serious infusion reactions such as anaphylaxis, convulsions, erythematous rash, and hypotension have occurred in <1% of patients. Delayed type HSR was detected in approximately 1% of patients. This usually occurred within 2 weeks of re-infusion, and was a combination of fever and/or rash symptoms with arthralgia and/or myalgia. These findings have been classified and reported as serum disease.

In a study conducted by Vultaggio et al., it was reported that IgE or IgM antibodies against infliximab could be detected and may play a role in IgE- and non-IgE-mediated anaphylaxis [132]. Skin testing and infliximab-specific antibody detection have been shown to be useful in predicting and preventing severe drug-related reactions [133].

### Certolizumab

Certolizumab is a subcutaneously administered selective immunosuppressive and anti-inflammatory TNF-α-inhibitor used to treat rheumatoid and psoriatic arthritis, spondyloarthritis, and Crohn’s disease. It is a mAb that binds to and blocks the activity of the proinflammatory cytokine TNF‑α.

In the EMA 2015 assessment report, mild to moderate ISRs were reported in 6.4% of patients, injection-related acute systemic HSR (pre-syncope) was reported in 0.2%, and delayed HSR was seen in 1.1% of patients [134].

Recently published FDA labels have reported that HSRs may rarely occur and include allergic dermatitis, rash, angioedema, dizziness, pyrexia, flush, ISRs, malaise, serum sickness, hypotension, dyspnea, and vasovagal syncope [135]. It is noted that some of these reactions were observed after the first administration. ISRs were reported in 1.7–3.2% of patients.

Although the literature contains no clear information on rates of hypersensitivity and anaphylaxis, it is stated that no single-drug related anaphylactic shock was observed in the one study conducted in 2013 [136].

### Rituximab

Rituximab is an intravenously delivered mAb that selectively binds to the CD20 antigen of B lymphocytes. Indications for its use include non-Hodgkin’s lymphoma, rheumatoid arthritis, and chronic lymphocytic leukemia.

Maggi et al. have reported acute infusion reaction frequencies of 10–77% during the first infusion (referenced in [65]). The FDA drug label for subcutaneous use [137] reports a 16–26% rate of local ISRs (pain, swelling, induction, hemorrhage, rash, pruritus, and erythema). Incidence rates reportedly decrease subsequent to the initial injection. The label also states that severe cytokine release syndrome and its symptoms (fever, tremor, urticaria, angioedema, bronchospasm, and severe dyspnea) may be seen within 1–2 h of infusion. However, the ratio is not specified in this label.

The recent FDA drug label for intravenous use [138] reports a ≥25.0% rate of IRs that typically (77%) occurred at the first infusion with the time onset between 30–120 min. This rate decreased after each ongoing infusion. Severe IRs may include symptoms such as acute respiratory distress syndrome, anaphylactic/anaphylactoid events, angioedema, bronchospasm, cardiogenic shock, hypoxia, hypotension, myocardial infarction, pulmonary infiltrates, urticaria, ventricular fibrillation, and death.

According to the BCCA drug assessment report (referenced in [139]) HSRs occurred in 1–10.0% of patients, IRs in 14–77.0% (severe, 0–7.0%), ISRs in 20.0%, and urticaria in 7.0% (severe, 1.0%) of patients. ISRs include local erythema, hemorrhage, induration, pain, pruritus, rash, and swelling at the injection site.

In a study conducted in 2009, the presence of serum anti-rituximab antibodies was reported in some patients in association with a less favourable treatment outcome [140]. Vultaggio and colleagues have shown that rituximab plays a role in specific T‑helper 2 cell and IgE responses in acute infusion reactions in addition to cytokine release mechanisms [141].

## Transplantation

A less frequent use of BSs is to minimize the rejection of transplanted organs (mostly kidneys, hearts, and livers). Basiliximab, muromonab, and daclizumab have been developed for this purpose. Today, only basiliximab is approved for this application. The other two drugs have been withdrawn from the market for a variety of reasons. Details about the reactions are given in Table 6.

**Table 6:** Reported allergic reactions to biotechnological substances (Transplantation and Various)

Biologics	ROA	Feature	Author	Year	HSR %	IR %	ISR %	Urticaria %	Anaphylaxis %
*Transplantation*									
Basiliximab	i.v.	Chimeric	FDA [142]	2001	3.0–10.0	–	–	–	–
			Erickson et al. [143]	2010	–	–	4.3	–	–
Belatacept	i.v.	Human	FDA [146]	2017	0	5.0	–	–	0
			EMA [147]	2019	–	4.4–5.5		–	–
Muromonab	s.c.–i.v.	Mouse	Withdrawn	–	–	–	–	–	–
Daclizumab	s.c.	Humanized	Withdrawn	–	–	–	–	–	–
*Various*									
Abciximab	i.v. s.c.	Chimeric	Dery et al. [150]	2004	0.3–0.6	–	–	0.1	0
			FDA [152]	2019	–	–	0.1–3.6	–	0
Eculizumab	i.v.	Humanized	FDA [153]	2019	–	–	–	–	–
			EMA [154]	2019	0.1–1.0	0.1–1.0		0.1–1.0	0.1–1.0
Lanadelumab	s.c.	Human	Banerji et al. [156]	2018	1.2	–	52.4	–	–
			EMA [157]	2018	1.2	–	53.0	–	0.0
Natalizumab	i.v.	Humanized	Maggi et al. [65]	2011	–	1.0–4.0	–	–	–
			EMA [158]	2016	<4.0	23.1		–	<1.0
			FDA [159]	2019	1.0–1.5	11–24.0		1.0–2.0	<1.0
Palivizumab	i.m.	Humanized	FDA [160]	2009	–	–	–	–	<0.001
			Chen et al. [161]	2015	0.05	–	–	–	–

Side effects and reactions are given as described in the literature

*ROA* *route of administration, s.c.* Subcutaneous, *i.v.* intravenous, *i.m.* intramuscular, *HSR* hypersensitivity reaction, *IR* Infusion reactions, *ISR* Injection-site reaction, *ADA* anti-drug antibody

### Basiliximab

Basiliximab is an immunosuppressive intravenously administered chimeric (75% human, 25% murine) mAb. It is used in combination with other immunosuppressive drugs to prevent acute graft rejection following renal transplantation. It acts by binding the alpha chain of the IL‑2 receptor on the surface of T lymphocytes.

The FDA drug label reports a >10.0% rate of side effects including nausea, vomiting, fever, dyspnea, and hypertension, and 3.0–10.0% of side effects include angina pectoris, cardiac failure, tachycardia, dizziness, bronchospasm, pulmonary edema, rhinitis, rash, and pruritus [142]. More detailed information is not provided. In a study conducted in 2010, infusion-related ISRs (pain, swelling, erythema) were found in 4.3% of patients [143]. In post-marketing surveys, HSRs have been reported in patients undergoing basiliximab therapy, including one case of IgE-mediated anaphylaxis [144].

Data on this BS are relatively limited, but according to the reports it seems to be mainly well tolerated by the patients [145]. Allergic reactions and anaphylaxis do not seem to be easily identified, since the patients experience intense immunosuppressive effects due to the concomitant medication. It does not induce CRS like muromonab-CD3, and its side effects are reported to be similar to placebo.

### Belatacept

Belatacept is a human CTLA-4/human IgG1 fusion protein that selectively inhibits T cell activity by binding to CD80 and CD86 of the antigen presenting cells.

According to the FDA 2017 labels [146], no patient had developed anaphylaxis or HSRs at the 3‑year follow-up. However, milder IRs (5%) were reported, similar to placebo, within 1 h of infusion. In post-marketing experiences, a case of anaphylaxis has been reported.

In the EMA 2019 assessment report [147], acute infusion events (flushing, headache, hypotension, hypertension) were reported in 4.4–5.5% of patients. Symptoms of diarrhea and pyrexia (≥2%), as well as hypertension, peripheral edema, nausea, and vomiting (≥20%), which may be “possible peri-infusional events,” were reported in 3‑year follow-up of the patients. There was no association found between these reactions and belatacept ADAs. Anaphylactic events have been observed in the post-marketing period, but no incidence was given.

### Muromonab

Muromonab is an immunosuppressive mAb given to reduce acute rejection in patients with kidney, liver, or heart transplants. It exerts its effect by targeting the CD3 receptor, a membrane protein expressed on the surface of T cells. Developments and progress of medical treatment in transplant medicine led to its withdrawal in 2010.

The literature contains information on cytokine release syndrome and anaphylaxis. In a book on this subject, symptoms of anaphylaxis such as pyrexia (90%; 40.0 °C or higher in 19% of cases), chills (59%), dyspnea (21%), nausea and vomiting (19%), chest pain (14%), diarrhea (14%), tremor (13%), bronchospasm (13%), headache (11%), tachycardia (10%), stiffness (8%), and hypertension (8%) were reported [148]. Pilot studies of this drug have been performed orally in patients with moderate to severe ulcerative colitis [149]. In this study, no severe side effects or CRS have been reported.

### Daclizumab

Daclizumab is a subcutaneously administered, immunosuppressive mAb used to treat relapsing-remitting multiple sclerosis and to prevent rejection of transplanted kidneys. The antibody reduces the number of circulating activated T cells by binding to the alpha subunit of the IL‑2 receptor on T lymphocytes and inhibiting interaction with IL‑2. In 2018, the drug was withdrawn from the market owing to reports of severe hepatotoxic side effects and encephalopathies.

## Other

Other indications for the use of biologicals include multiple sclerosis, paroxysmal nocturnal hemoglobinuria, instable angina pectoris during insertion of cardiac catheters, congenital bronchopulmonary dysplasia, and congenital heart disease.

### Abciximab

Abciximab is an intravenously administered chimeric mAb fragment that inhibits binding of platelet adhesion proteins (such as fibrinogen, fibronectin, von-Willebrand-Factor) to glycoprotein IIb/IIIa receptors on the surface of platelets. It is frequently used to prevent ischemic complications associated with invasive heart procedures, as well as to prolong cardiac infarct prophylaxis in patients with unstable angina pectoris due to its antithrombotic effect.

The registry study of the drug reported hypersensitivity rates of 0.3–0.6%, an urticaria rate of 0.1%, and no cases of anaphylaxis or angioedema [150]. In the same study, human anti-chimeric antibodies were found to be associated with thrombocytopenia. The literature contains a very small number of case reports. One developed an anaphylaxis 2–4 h after treatment [151]. The FDA label reported bronchospasm (0.3%), injection site pain (0.1–3.6%), and pruritus (0.5%) [152]. No incidence of anaphylaxis has been reported in any of the phase III studies (clinical trials).

### Eculizumab

Eculizumab is the humanized mAb used primarily for the treatment of paroxysmal nocturnal hemoglobinuria and atypical haemolytic uremic syndrome. It can also be used in the treatment of generalized myasthenia gravis and neuromyelitis optica spectrum disorder with antibody positivity. This drug acts by binding to the C5 component of the complement system, inhibiting its activation, and consequently contributes to reducing erythrocyte destruction and hemolysis.

FDA labels reported that IRs, HRs, and anaphylaxis may develop during treatment, but IRs requiring discontinuation of the drug have not been reported [153]. In the EMA 2019 assessment report, HSRs, IRs, urticaria, and anaphylaxis are stated as uncommon (≥1/1000–<1/100) [154].

### Lanadelumab

Lanadelumab is a human mAb (class IgG1 kappa) that specifically inhibits the activity of plasma kallikrein, used to reduce attacks and representing a prophylaxis measure in patients with hereditary angioedema [155].

In a study published in 2018, 52.4% of patients reported ISRs (34.1% in the placebo group) with symptoms such as bruising, dizziness, erythema, and local pain [156]. Only one patient (1.2%) developed mild-to-moderate HSRs, which included transient symptoms of oral tingling and pruritus, and resolved spontaneously within 1 day after onset without need for medication.

The EMA 2018 evaluation report [157] provides more detailed data on the study conducted by Banerji et al. [156]. In this report, ISRs are given as follows; pain (41.7%), erythema (9.5%), bruising (6.0%), discomfort (3.6%), hemorrhage (3.6%), pruritus (3.6%), swelling (3.6%), hematoma (2.4%), induration (2.4%), paresthesia (2.4%), warmth (2.4%), edema (1.2%), and rash (1.2%). In addition, no events of anaphylactoid reaction or anaphylaxis were reported (referenced in [157]).

### Natalizumab

Natalizumab is an intravenously administered, immunosuppressive recombinant humanized IgG4 antibody that is produced in mouse cells. It acts by binding integrin a4 and is used to treat relapsing-remitting multiple sclerosis.

Maggi and colleagues (referenced in [65]) reported IRs in 1–4% of the patients in their review.

According to the EMA 2016 evaluation report [158], 23.1% of patients experienced infusion-related reactions, HSRs occurred in up to 4% of patients, and anaphylactic/anaphylactoid reactions in less than 1%. HSRs usually occurred during infusion or within 1 h after completion. Hypersensitivity symptoms included rash and urticaria, hypotension, hypertension, chest pain, chest complaints, dyspnea, and angioedema.

The FDA’s 2019 label reported infusion-related reactions in 11–24% of patients (placebo 7–18.0%), acute urticaria in 1–2%, acute HSRs in 1.5%, pruritus in 4%, and serious IRs and anaphylaxis in <1% [159]. Symptoms frequently associated with these reactions were fever, chills, rash, urticaria, pruritus, dizziness, nausea, flush, hypotension, dyspnea, and chest pain. The reactions occurred mostly within 2 h after infusion.

### Palivizumab

Palivizumab is an intramuscularly administered humanized mAb (95% human and 5% murine) that is used in pediatric patients to prevent respiratory syncytial virus (RSV) infections. The antibody binds to coat proteins on the viral surface, thereby inhibiting its entry into host cells. It is used in patients with bronchopulmonary dysplasia, cystic fibrosis, and neonatal heart disease that are considered to be at high risk of severe RSV-associated disease.

Serious acute HSRs were rare among the 400,000 patients treated to date (>2 million doses) [160]. Anaphylactic reactions to repeated dosing or during the follow-up period (<1 case per 100,000 patients) have also been very rare [160]. HSRs include dyspnea, respiratory insufficiency, cyanosis, urticaria, pruritus, angioedema, hypotonia, and unresponsiveness. No fatal reactions have been observed in any patient.

In a study conducted by Chen et al. [161] on 13,025 infants (that received 57,392 injections), HSR was observed at a rate of 0.05%. In another study, injections were generally well tolerated, and drug-associated side effects were observed at a rate of 3.4% [162].

## Discussion

**Fig. 1:**
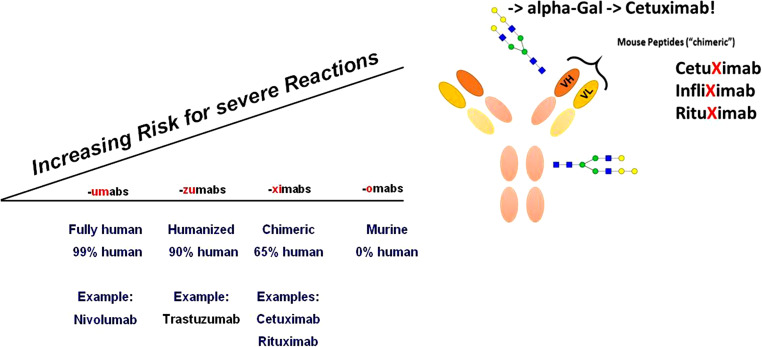
Risk assessment of biotechnological substances

BSs are increasingly used today and play an important role in the treatment of allergic, pulmonary, dermatological, oncological, rheumatological, as well as various other diseases. These drugs can be used as first-line or second-line treatment, as well as for prevention purposes. Observed HSRs may be mild, moderate, but may also be life-threatening by severely affecting vital parameters. All organ systems can be affected during reactions, so clinical symptoms are quite diverse. Reactions may occur due to allergic, non-allergic, or immunological side effects. The risk of allergic reactions decreases proportionally with the increase in human homology of the BS (Fig. 1; [163]). Vultaggio and co-authors explain the reactions to BSs with two possible main mechanisms: (i) loss of immune tolerance due to side effect of the BSs; and (ii) triggering of immune responses and reactions to non-self epitopes in BSs [164].

This comprehensive review reveals a hypersensitivity prevalence against various BSs and an inconsistency and lack of precision regarding the nomenclature used for immune reactions to BSs (hypersensitivity, anaphylaxis, anaphylactoid, and infusion reactions) in the literature. Theoretically defined hypersensitivity and the hypersensitivity that is observed and classified in clinical practice seem to be different. Similarly, many data reported as anaphylaxis actually describe severe anaphylactic reactions (grades III or IV). It became evident that these definitions describe many common symptoms. Due to the overlap in signs and symptoms in the reported descriptions, it is not always possible to differentiate these reactions properly according to their pathomechanism. The common points of all these reactions are indicated in Table 7 [165–167].

**Table 7:** Grading of hypersensitivity reactions, anaphylaxis, acute infusion reactions, and cytokine-release syndrome

Reaction	Grade I	Grade II	Grade III	Grade IV	Grade V
HSR [165]	Transient flush, rash or exanthema, drug-induced fever <38 °C	Flush, rash, exanthema, urticaria, and dyspnea, drug-induced fever ≥38 °C	Symptomatic bronchospasm with or without urticaria, hypotension, and angioedema	Anaphylaxis	Death
Anaphylaxis [166]	Pruritus, flush, urticaria, angioedema	Pruritus, flush, urticaria, angioedema, nausea, cramping, rhinorrhea, hoarseness, dyspnea, tachycardia, blood pressure change, arhythmia, vomiting	Pruritus, flush, urticaria, angioedema, vomiting, defecation, laryngeal edema, bronchospasm, cyanosis, shock	Pruritus, flush, urticaria, angioedema, vomiting, defecation, respiratory arrest, cardiac arrest	Death
Acute IR [165]	Mild reaction	Requires therapy interruption, prophylactic medication indicated for ≥24 h	Prolonged symptoms or not rapidly responsive to intensive medication	Life-threatening Need for vasopressor or ventilatory support	Death
	Grade I and II reactions: chills, dizziness, dyspnea, fever, flushing, headache, myalgia, rigors, and mild hypotension		Grades III and IV: anaphylaxis, bronchospasms, cardiac dysfunction, severe hypotension requiring medication, and other symptoms		
CRS [167]	Fever, constitutional symptoms	Hypotension responding to fluids or low dose vasopressors, grade 2 organ toxicities	Schock requiring high dose/multiple vasopressors, Hypoxia requiring ≥ 40% Fractional Inspired O_2_ Concentration (FiO_2_), grade 3 organ toxicities, grade 4 transaminases	Mechanical ventilation, grade 4 organ toxicities (excl. transaminases)	–
	Mild cases may show flu-like symptoms. Grade III and IV shows life-threatening hematologic, cardiovascular, neurological, pulmonary, and renal involvement and symptoms. *Unspecific: *anorexia, fever, fatigue, nausea, vomiting *Extremity: *arthralgia, edema, myalgia, rash, and rigor *Gastrointestinal: *diarrhea, hepatomegaly, splenomegaly *Hematologic: *coagulopathy, elevated liver enzyme, febrile neutropenia, disseminated intravascular coagulation, liver failure *Neurologic: *aphasia, confusion, headache, delirium, paresis, seizures *Respiratory: *hypoxia, pulmonary edema, respiratory failure, tachypnea *Cardiac: *acute heart failure, arrhythmia, hypotension, stress cardiomyopathy, tachycardia, QT prolongation *Renal: *acute kidney injury, acute renal failure				

*HSR* hypersensitivity reaction, *IR* infusion reaction, *CRS* cytokine release syndrome

**Fig. 2:**
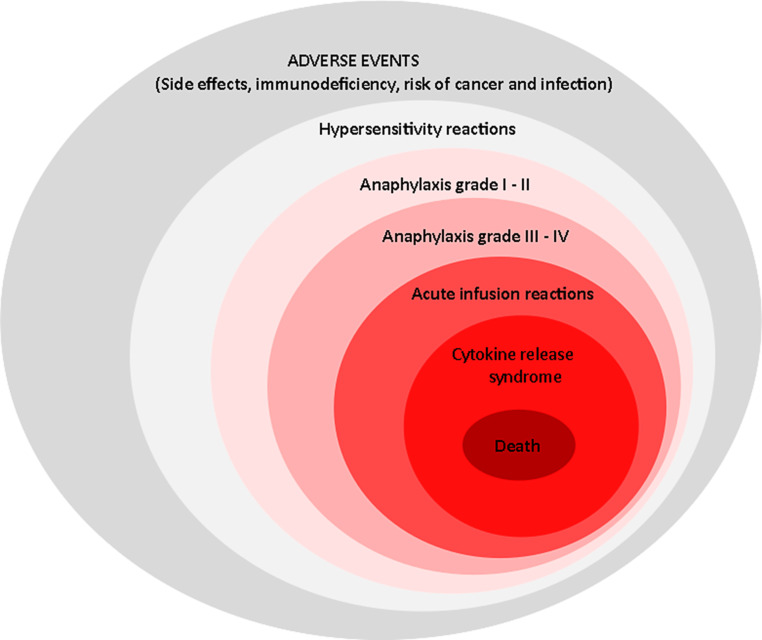
Synopsis of all reactions to biotechnological substances

**Fig. 3:**
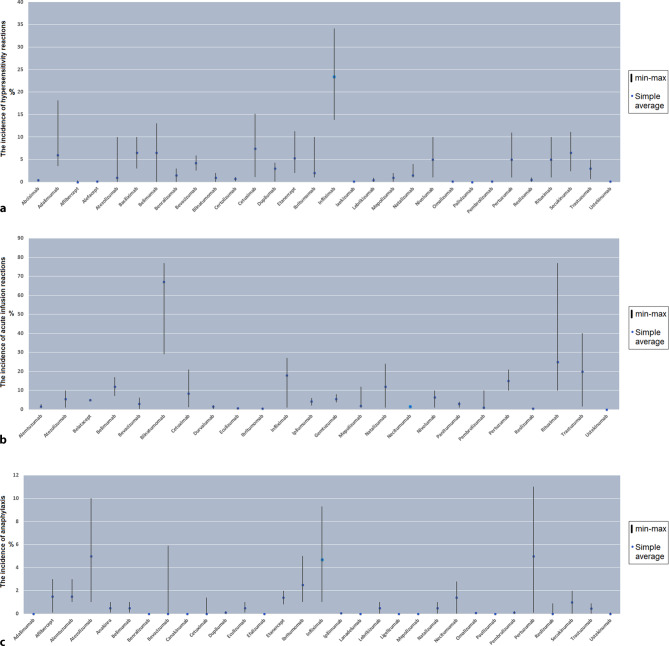
**a** A descriptive graph on the incidence of hypersensitivity reactions to biotechnological substances according to the reviewed databases (for references see the description of the respective BS in the text). **b** A descriptive graph on the incidence of acute infusion reactions to biotechnological substances according to the reviewed databases (for references see the description of the respective BS in the text). **c** A descriptive graph on the incidence of anaphylaxis to biotechnological substances according to the reviewed databases (for references see the description of the respective BS in the text)

In addition, reactions such as pruritus, flush, and urticaria are also included in the definitions of IR, HSR, anaphylaxis, and CRS. A summary view of the reactions according to severity is shown in Fig. 2. Fig. 3 shows a synopsis of the incidences of HSRs (Fig. 3a), acute IRs (Fig. 3b), and anaphylaxis to BSs (Fig. 3c) as extracted by the authors from the comprehensive review of the databases. There is an urgent need to define and classify these reactions more precisely in the future. Furthermore, the development of a scoring system, including symptom- or system-based approaches, can make it easier for us to identify the reactions of our patients and treat them adequately. This scoring can be evaluated according to which organ system (cardiac, gastrointestinal, hematologic, muscular, neurological, renal, respiratory, skin, vascular) is affected and the severity of symptoms.

One of the important points that may have been overlooked is the understanding of whether some symptoms (such as nasopharyngitis, conjunctivitis, rhinitis, thrombocytopenia, anemia, or gastrointestinal symptoms, etc.) have an immunological origin. The role of ADAs in these symptoms is not yet known. In addition, the risk of patients at each injection/infusion should be defined according to real life. In general, the literature gives percentages by calculation and only follow-up reactions in the treatment process. The long-term evaluations seem to be rather small in number.

CRS, which is a form of severe IR, can also be observed during alemtuzumab, anti-CD28 monoclonal antibody TGN1412, anti-thymocyte globulin, blinatumomab, brentuximab, dacetuzumab, muromonab-CD3, nivolumab, rituximab, and obinutuzumab treatments (referenced in [167]). A large majority of patients may have sepsis/septic shock fragments, as well as symptoms that may also be confused with tumor lysis syndrome. IL‑6 plays an important role in the development of CRS; therefore siltuximab or tocilizumab, which is a chimeric mAb against IL‑6 and receptors, is used for the treatment of this condition [167]. In cases where CRS developed during treatment, the relationship between the drug itself and ADA is so far unknown.

## Conclusion

BSs are important immunotherapeutic tools used in the treatment of many diseases today. Various side effects and reactions can be observed during treatment, but there are deficiencies in their identification and classification. During this comprehensive research, it became evident that a more precise nomenclature should be applied. In order to achieve a harmonization in this regard, a simpler symptom- or system-based classification and scoring system is needed. A better understanding of the pathophysiology of hypersensitivity reactions and increased clinical experience in the treatment of side effects will provide timely control of unexpected reactions. As a result, immunotherapy with BSs will become safer in the future.

## Abbreviations

α‑Gal: Galactose-α‑1,3‑galactose
ADA: Anti-drug antibodies
ADR: Adverse drug reaction
ALL: Acute lymphoblastic leukemia
AML: Acute myeloid leukemia
ARCN: Airway Research Center North
BCCA: British Columbia Cancer Agency
BLyS: B lymphocyte stimulant
BMBF: Federal Ministry for Science and Education
BS: Biotechnological substance
CAPS: Cryopyrin-associated periodic syndromes
COPD: Chronic obstructive pulmonary disease
CRS: Cytokine release syndrome
CTLA‑4: Cytotoxic T lymphocyte-associated antigen 4
EAACI: European Academy of Allergy and Clinical Immunology
EGFR: Epidermal growth factor receptors
EMA: European Medicines Agency
Fab: Antigen-binding fragment
FDA: Food and Drug Administration
FMF: Familial Mediterranean fever
HAMA: Human anti-murine antibodies
HER2: Human epidermal growth factor receptor 2 protein
HSR: Hypersensitivity reaction
IgE: Immunoglobulin E
IL: Interleukin
IPF: Idiopathic pulmonary fibrosis
IR: Infusion reaction
ISR: Injection-site reaction
LFA‑1: Lymphocyte function-associated antigen‑1
mAb: Monoclonal antibody
PD-L1: Programmed death ligand 1
RA: Rheumatoid arthritis
SC: Subcutaneous
SmPC: Summary of product characteristic
TEAE: Treatment-emergent adverse event
TGF: Transforming growth factor
TNF: Tumor necrosis factor
TRAPS: Tumor necrosis factor receptor-associated periodic syndrome
VEGF: Vascular endothelial growth factor
